# Supplementary material for: Engineering eukaryote-like regulatory circuits to expand artificial control mechanisms for metabolic engineering in Saccharomyces cerevisiae
Source: Commun Biol. 2022 Feb 16;5:135. doi: 10.1038/s42003-022-03070-z (PMC8850539; doi:10.1038/s42003-022-03070-z)
Supplement: Supplementary file 2 — Supplementary Information [file 42003_2022_3070_MOESM2_ESM.pdf]

Supplementary information for:

Engineering eukaryote-like regulatory circuits to expand artificial control mechanisms for metabolic engineering in *Saccharomyces cerevisiae*

Bingyin Peng [1, 2, 3, 4]*, Naga Chandra Bandari [1], Zeyu Lu [1], Christopher B. Howard [1], Colin Scott [2,5], Matt Trau[1, 6], Geoff Dumsday [7], Claudia E. Vickers [2,3,4,8]*

[1] Australian Institute for Bioengineering and Nanotechnology (AIBN), The University of Queensland, Brisbane, QLD 4072, Australia

[2] CSIRO Future Science Platform in Synthetic Biology, Commonwealth Scientific and Industrial Research Organisation (CSIRO), Black Mountain, ACT 2601, Australia

[3] ARC Centre of Excellence in Synthetic Biology, Queensland University of Technology, Brisbane, QLD 4000, Australia

[4] Centre for Agriculture and the Bioeconomy, School of Biological and Environmental Science, Queensland University of Technology, Brisbane, QLD 4000, Australia

[5] Biocatalysis and Synthetic Biology Team, CSIRO Land & Water, Black Mountain Science and Innovation Park, Canberra, ACT 2601, Australia

[6] School of Chemistry and Molecular Biosciences (SCMB), The University of Queensland, Brisbane, QLD 4072, Australia

[7] CSIRO Manufacturing, Clayton, VIC 3169. Australia

[8] Griffith Institute for Drug Discovery, Griffith University, Brisbane, QLD 4111, Australia

* Corresponding authors

Claudia E. Vickers (claudia.vickers@csiro.au)

Bingyin Peng (bingyin.peng@qut.edu.au)

Supplementary Table 1. Plasmids used in this work

Plasmid	Properties	Reference
pILGFP1D5	Yeast integration plasmid; P_{URA3}>*KlURA3*>T_{AgTEF1}-*(BamHI)yEGFP*>T_{PGK1}-T_{URA3}	[1]
pILGFP1F5	Yeast integration plasmid; P_{URA3}>*KlURA3*>T_{AgTEF1}-P_{TEF1}>*(BamHI)yEGFP*>T_{PGK1}-T_{URA3}	[1]
pILGFP1H5	Yeast integration plasmid; P_{URA3}>*KlURA3*>T_{AgTEF1}-P_{TEF1}>*yEGFP*>T_{PGK1}-T_{URA3}	[1]
pILGFP1E8	Yeast integration plasmid; P_{URA3}>*KlURA3*>T_{AgTEF1}-P_{GLN3}>*yEGFP*>T_{PGK1}-T_{URA3}	This work
pILGFP1G8	Yeast integration plasmid; P_{URA3}>*KlURA3*>T_{AgTEF1}-P_{TOR1}>*yEGFP*>T_{PGK1}-T_{URA3}	This work
pILGFP1A9	Yeast integration plasmid; P_{URA3}>*KlURA3*>T_{AgTEF1}-P_{DAL80}>*yEGFP*>T_{PGK1}-T_{URA3}	This work
pILGFP1C9	Yeast integration plasmid; P_{URA3}>*KlURA3*>T_{AgTEF1}-P_{GCR1}>*yEGFP*>T_{PGK1}-T_{URA3}	This work
pILGFP1E9	Yeast integration plasmid; P_{URA3}>*KlURA3*>T_{AgTEF1}-P_{GCR2}>*yEGFP*>T_{PGK1}-T_{URA3}	This work
pILGFP1G9	Yeast integration plasmid; P_{URA3}>*KlURA3*>T_{AgTEF1}-P_{SNF1}>*yEGFP*>T_{PGK1}-T_{URA3}	This work
pILGFP1AA	Yeast integration plasmid; P_{URA3}>*KlURA3*>T_{AgTEF1}-P_{YPK2}>*yEGFP*>T_{PGK1}-T_{URA3}	This work
pILGFP1CA	Yeast integration plasmid; P_{URA3}>*KlURA3*>T_{AgTEF1}-P_{ADR1}>*yEGFP*>T_{PGK1}-T_{URA3}	This work
pILGFP1EA	Yeast integration plasmid; P_{URA3}>*KlURA3*>T_{AgTEF1}-P_{NRG1}>*yEGFP*>T_{PGK1}-T_{URA3}	This work
pILGFP1GA	Yeast integration plasmid; P_{URA3}>*KlURA3*>T_{AgTEF1}-P_{MIG1}>*yEGFP*>T_{PGK1}-T_{URA3}	This work
pILGFP1CB	Yeast integration plasmid; P_{URA3}>*KlURA3*>T_{AgTEF1}-P_{ROX1}>*yEGFP*>T_{PGK1}-T_{URA3}	This work
pILGFP1EB	Yeast integration plasmid; P_{URA3}>*KlURA3*>T_{AgTEF1}-P_{HAP4}>*yEGFP*>T_{PGK1}-T_{URA3}	This work
pILGFP1GB	Yeast integration plasmid; P_{URA3}>*KlURA3*>T_{AgTEF1}-P_{HAC1}>*yEGFP*>T_{PGK1}-T_{URA3}	This work
pILGFP4D5	Yeast integration plasmid; P_{URA3}>*KlURA3*>T_{AgTEF1}-P_{UPC2}>*yEGFP*>T_{PGK1}-T_{URA3}	This work
pILGFP2BC9	Yeast integration plasmid; P_{URA3}>*KlURA3*>T_{AgTEF1}-$P_{TEF1+[Adaptor]}$>*yEGFP*>T_{PGK1}-T_{URA3}	This work
pILGFP2H8	Yeast integration plasmid; P_{URA3}>*KlURA3*>T_{AgTEF1}-$P_{CYC1+MalO}$>*yEGFP*>T_{PGK1}-T_{URA3}	This work
pILGFP3E9	Yeast integration plasmid; P_{URA3}>*KlURA3*>T_{AgTEF1}-$P_{CYC1+[Adaptor]}$ >*yEGFP*>T_{PGK1}-T_{URA3}<br><br>Note: $P_{CYC1+[Adaptor]}$ refers to P15 core promoter [2]	This work
pILGFP4D41	Yeast integration plasmid; P_{URA3}>*KlURA3*>T_{AgTEF1}-$P_{TEF1+[Z268]}$>*yEGFP*>T_{PGK1}-T_{URA3}	This work

pILGFP4D42	Yeast integration plasmid; P_{URA3}>*KlURA3*>T_{AgTEF1}-$P_{CYC1+[Z268]}$>*yEGFP*>T_{PGK1}-T_{URA3}	This work
pILGFP8H8	Yeast integration plasmid; P_{URA3}>*KlURA3*>T_{AgTEF1}-$P_{TEF1+[PZ4]}$>*yEGFP*>T_{PGK1}-T_{URA3}	This work
pRS414	Yeast centromere plasmid; *CEN6_ARS4 TRP1*	[3]
pAF4EH4	pRS414; P_{HAC1}>*Zif268*>*VP16*-P_{ABF1}	This work
pAF4EH4R	pRS414; P_{HAC1}>*Zif268*>*VP16*>P_{ABF1}	This work
pAF5HB	pRS414; P_{HAC1}>*Zif268*>*10*WD*>P_{ABF1}	This work
pAF7C1	pRS414; P_{HAC1}>*Zif268*>*MED3*>P_{ABF1}	This work
pAF7E1	pRS414; P_{HAC1}>*Zif268*>*MED15*>P_{ABF1}	This work
pAF7G1	pRS414; P_{HAC1}>*Zif268*>$GCN4^{A}$>P_{ABF1}	This work
pAF7A2	pRS414; P_{HAC1}>*Zif268*>$SIN3^{C}$>P_{ABF1}	This work
pAF7C2	pRS414; P_{HAC1}>*Zif268*> *TUP1*>P_{ABF1}	This work
pAF7E2	pRS414; P_{HAC1}>*Zif268*>$MIG1^{C}$>P_{ABF1}	This work
pILGFP9E3	Yeast integration plasmid; P_{URA3}>*KlURA3*>T_{AgTEF1}-$P_{TEF1+[TetO]}$>*yEGFP*>T_{PGK1}-T_{URA3}	This work
pAF9C3	pRS414; P_{HAC1}>*RevTetR_r1.7*>$SIN3^{C}$>P_{ABF1}	This work
pAF4E9D	pRS414; P_{HAC1}>*LacI*>*TUP1*>P_{ABF1}	This work
pAF9D91	pRS414; P_{HAC1}>*TetR*>$SIN3^{C}$>P_{ABF1}	This work
pAF9D92	pRS414; P_{HAC1}>*TetR*>*TUP1*>P_{ABF1}	This work
pAF9D93	pRS414; P_{HAC1}>*TetR*>$MIG1^{C}$>P_{ABF1}	This work
pAF9D94 (pAF10C6)	pRS414; P_{HAC1}>*TetR*>*CYC8*>P_{ABF1}	This work
pAF8A5B	pRS414; P_{HAC1}>*ZifPZ42*>*MED3*>P_{ABF1}	This work
pAFZifPZ421	pRS414; P_{HAC1}>*ZifPZ42*>*MED3*>P_{ABF1}	This work
pAFZifPZ431	pRS414; P_{HAC1}>*ZifPZ43*>*MED3*>P_{ABF1}	This work
pAFZifPZ441	pRS414; P_{HAC1}>*ZifPZ44*>*MED3*>P_{ABF1}	This work
pAFTALPZ41	pRS414; P_{HAC1}>*TALPZ4*>*MED3*>P_{ABF1}	This work
pAF9H6	pRS414; P_{HAC1}>*TALPZ4*>*MED15*>P_{AFB1}	This work
pAFZifPZ422	pRS414; P_{HAC1}>*ZifPZ42*>*MED15*>P_{AFB1}	This work
pAFZifPZ432	pRS414; P_{HAC1}>*ZifPZ43*>*MED15*>P_{AFB1}	This work
pAFZifPZ442	pRS414; P_{HAC1}>*ZifPZ44*>*MED15*>P_{AFB1}	This work
pILGFP3A2 (1)	Yeast integration plasmid; P_{URA3}>*KlURA3*>T_{AgTEF1}-P_{TEF1}>*2*TcRb*>*yEGFP*>T_{PGK1}-T_{URA3}<br><br>Note: TcRB, Tetracycline riboswitch	This work
pILGFP3A2 (2)	Yeast integration plasmid; P_{URA3}>*KlURA3*>T_{AgTEF1}-P_{TEF1}>*3*TcRb*>*yEGFP*>T_{PGK1}-T_{URA3}	This work
pIR3DH8	Yeast integration plasmid; *gal80Arm1*-P_{AgTEF1}-*KlURA3*-T_{AgTEF1}-*gal80Arm2*	[1]
pUG6	*loxP*-P_{AgTEF1}-*kanMX*-P_{AgTEF1}-*loxP*	[4]
pIRTetR-GAL80	*gal80Arm1*-P_{AgTEF1}-*KanMX4*-T_{AgTEF1}-P_{HAC1}>*TetR*>*TUP1*>P_{ABF1}-$P_{TEF1+[TetO]}$>*GAL80Arm2*	This work

pILGFP8EFA	Yeast integration plasmid; P_{URA3}>KlURA3>T_{AgTEF1}-P_{TEF1}>UBI4>RHGSGTMV>DHFR^{P66L}>yEGFP>T_{PGK1}-T_{URA3}	This work
pILGFP4M	Yeast integration plasmid; P_{URA3}>KlURA3>T_{AgTEF1}-P_{SeGAL2}>(BamHI)yEGFP>T_{URA3}	[5]
pILGFP4Q	Yeast integration plasmid; P_{URA3}>KlURA3>T_{AgTEF1}-P_{SkGAL2}(1*PZ4)>(BamHI)yEGFP>T_{URA3}	[5]
pILGFP10E1A	Yeast integration plasmid; P_{URA3}>KlURA3>T_{AgTEF1}-P_{SkGAL2}M1(2*PZ4)> (BamHI)yEGFP>T_{URA3}	This work
pILGFP10E1B	Yeast integration plasmid; P_{URA3}>KlURA3>T_{AgTEF1}-P_{SkGAL2}M2(3*PZ4)>(BamHI)yEGFP>T_{URA3}	This work
pILGFP10CF72	Yeast integration plasmid; P_{URA3}>KlURA3>T_{AgTEF1}-P_{SkGAL2}M4(4*PZ4)>(BamHI)yEGFP>T_{URA3}	This work
pILGFP10CF72 (B9)	Yeast integration plasmid; P_{URA3}>KlURA3>T_{AgTEF1}-P_{SkGAL2}M5(5*PZ4)>(BamHI)yEGFP>T_{URA3}	This work
pILGFP10CF72 (B11)	Yeast integration plasmid; P_{URA3}>KlURA3>T_{AgTEF1}-P_{SkGAL2}M3(2*PZ4)>(BamHI)yEGFP>T_{URA3}	This work
pILGFP10CF71	Yeast integration plasmid; P_{URA3}>KlURA3>T_{AgTEF1}-$P_{SkGAL2+[Z268]}$>(BamHI)yEGFP>T_{URA3}	This work
pUG66	loxP-P_{AgTEF1}-ble-P_{AgTEF1}-loxP	[7]
pJT9RFR	Yeast/E. coli shuttle plasmid; 2µ LEU2 T_{RPL3}>ERG20>P_{GAL1}-P_{GAL2}>Y-FAST>ErB1.2A-AcNES1>T_{RPL41B}	[1]

Supplementary Table 2. *Saccharomyces cerevisiae* strains used in this work

Strain	Genotype/Note	Reference
CEN.PK2-1C	MATa ura3-52 trp1-289 leu2-3,112 his3Δ 1	[8]
CEN.PK113-5D	MATa ura3-52	[8]
**ILHA series strains**		
GH4	CEN.PK113-5D derivative; *ura3(1, 704)::KlURA3>*T_{AgTEF1}	[6]
G89S	CEN.PK113-5D derivative; *ura3(1, 704):: KlURA3>*T_{AgTEF1}-P_{TEF1}*>(BamHI)yEGFP*	[6]
	Figure 1b, Construct 1	
G1F5	CEN.PK113-5D derivative; *ura3(1, 704):: KlURA3>*T_{AgTEF1}-P_{TEF1}*>(BamHI)yEGFP>*T_{PGK1}	[1]
	Figure 1b, Construct 2	
G1H5	CEN.PK113-5D derivative; *ura3(1, 704):: KlURA3>*T_{AgTEF1}-P_{TEF1}*>yEGFP>*T_{PGK1}	[1]
	Figure 1b: Construct 3<br>Figure 1b: Promoter_*TEF1*, Trans-factor_None	
G1E8	CEN.PK113-5D derivative; *ura3(1, 704):: KlURA3>*T_{AgTEF1}-P_{GLN3}*>yEGFP>*T_{PGK1}	This work
	Figure 1c: Promoter_*GLN3*	
G1G8	CEN.PK113-5D derivative; *ura3(1, 704):: KlURA3>*T_{AgTEF1}-P_{TOR1}*>yEGFP>*T_{PGK1}	This work
	Figure 1c: Promoter_*TOR1*	
G1A9	CEN.PK113-5D derivative; *ura3(1, 704):: KlURA3>*T_{AgTEF1}-P_{DAL80}*>yEGFP>*T_{PGK1}	This work
	Figure 1c: Promoter_*TOR1*<br>Figure 1d	
G1C9	CEN.PK113-5D derivative; *ura3(1, 704):: KlURA3>*T_{AgTEF1}-P_{GCR1}*>yEGFP>*T_{PGK1}	This work
	Figure 1c: Promoter_*GCR1*	
G1E9	CEN.PK113-5D derivative; *ura3(1, 704):: KlURA3>*T_{AgTEF1}-P_{GCR2}*>yEGFP>*T_{PGK1}	This work
	Figure 1c: Promoter_*GCR2*	
G1G9	CEN.PK113-5D derivative; *ura3(1, 704):: KlURA3>*T_{AgTEF1}-P_{SNF1}*>yEGFP>*T_{PGK1}	This work
	Figure 1c: Promoter_*SNF1*	
G1AA	CEN.PK113-5D derivative; *ura3(1, 704):: KlURA3>*T_{AgTEF1}-P_{YPK2}*>yEGFP>*T_{PGK1}	This work
	Figure 1c: Promoter_*YPK2*	
G1CA	CEN.PK113-5D derivative; *ura3(1, 704):: KlURA3>*T_{AgTEF1}-P_{ADR1}*>yEGFP>*T_{PGK1}	This work
	Figure 1c: Promoter_*ADR1*	
G1EA	CEN.PK113-5D derivative; *ura3(1, 704):: KlURA3>*T_{AgTEF1}-P_{NRG1}*>yEGFP>*T_{PGK1}	This work
	Figure 1c: Promoter_*NRG1*	
G1GA	CEN.PK113-5D derivative; *ura3(1, 704):: KlURA3>*T_{AgTEF1}-P_{MIG1}*>yEGFP>*T_{PGK1}	This work
	Figure 1c: Promoter_*MIG1*	
G1CB	CEN.PK113-5D derivative; *ura3(1, 704):: KlURA3>*T_{AgTEF1}-P_{ROX1}*>yEGFP>*T_{PGK1}	This work

	Figure 1c: Promoter_*ROX1*	
G1EB	CEN.PK113-5D derivative; *ura3(1, 704):: KlURA3>*T_{AgTEF1}-P_{HAP4}*>yEGFP>*T_{PGK1}<br>Figure 1c: Promoter_*HAP4*	This work
G1GB	CEN.PK113-5D derivative; *ura3(1, 704):: KlURA3>*T_{AgTEF1}-P_{HAC1}*>yEGFP>*T_{PGK1}<br>Figure 1c: Promoter_*HAC1*	This work
G5E4	CEN.PK113-5D derivative; *ura3(1, 704):: KlURA3>*T_{AgTEF1}-P_{UPC2}*>yEGFP>*T_{PGK1}<br>Figure 1c: Promoter_*UPC2*	This work
G2BC9	CEN.PK113-5D derivative; *ura3(1, 704):: KlURA3>*T_{AgTEF1}-$P_{TEF1(-)}$*>yEGFP>*T_{PGK1}<br>Figure 2b: Promoter_*TEF1*+, Trans-factor_None	This work
G3E9	CEN.PK113-5D derivative; *ura3(1, 704):: KlURA3>*T_{AgTEF1}-$P_{CYC1(-)}$*>yEGFP>*T_{PGK1}<br>Figure 1b: Promoter_*CYC1*+, Trans-factor_None	This work
G4D41	CEN.PK2-1C derivative; *ura3(1, 704):: KlURA3>*T_{AgTEF1}-$P_{TEF1+4\times[Z268]}$*>yEGFP>*T_{PGK1}	This work
G4D42	CEN.PK2-1C derivative; *ura3(1, 704):: KlURA3>*T_{AgTEF1}-$P_{CYC1+4\times[Z268]}$*>yEGFP>*T_{PGK1}	This work
G4D41A	G4D41 derivative; pRS414<br>Figure 2b	This work
G4D41C	G4D41 derivative; pAF4EH4R<br>Figure 2b Trans-factor_Zif268-VP16[A]	This work
G4D41D	G4D41 derivative; pAF5HB<br>Figure 2b Trans-factor_Zif268-10*WD	This work
G4D41E	G4D41 derivative; pAF7C1<br>Figure 2b Trans-factor_Zif268-Med3	This work
G4D41F	G4D41 derivative; pAF7E1<br>Figure 2b Trans-factor_Zif268-Med15	This work
G4D41G	G4D41 derivative; pAF7G1<br>Figure 2b Trans-factor_Zif268-Gcn4[A]	This work
G4D41J	G4D41 derivative; pAF7E2<br>Figure 2b Trans-factor_Zif268-Mig1[C]	This work
G4D42A	G4D42 derivative; pRS414<br>Figure 2a	This work
G4D42C	G4D42 derivative; pAF4EH4R<br>Figure 2a Trans-factor_Zif268-VP16[A]	This work
G4D42D	G4D42 derivative; pAF5HB<br>Figure 2a Trans-factor_Zif268-10*WD	This work
G4D42E	G4D42 derivative; pAF7C1<br>Figure 2a Trans-factor_Zif268-Med3	This work
G4D42F	G4D42 derivative; pAF7E1<br>Figure 2a Trans-factor_Zif268-Med15	This work
G4D42G	G4D42 derivative; pAF7G1<br>Figure 2a Trans-factor_Zif268-Gcn4[A]	This work
G4D42J	G4D42 derivative; pAF7E2<br>Figure 2a Trans-factor_Zif268-Mig1[C]	This work

G8H8	CEN.PK2-1C derivative; *ura3(1, 704)::KlURA3>*T_{AgTEF1}-$P_{TEF1+4\times[PZ4]}$*>yEGFP>*T_{PGK1}	This work
G8H8N	G8H8 derivative; pAFZifPZ431<br>Figure 2b Trans-factor_ZifPZ43-Med3	This work
G8H8O	G8H8 derivative; pAFZifPZ432<br>Figure 2b Trans-factor_ZifPZ43-Med15	This work
G9E3	CEN.PK2-1C derivative; *ura3(1, 704):: KlURA3>*T_{AgTEF1}-$P_{TEF1+4\times[TetO]}$*>yEGFP>*T_{PGK1}	This work
G9E3C	G9E3 derivative; pAF4EH4R<br>Figure 3b TetR-repressor_None	This work
G9E3R	G9E3 derivative; pAF9D91<br>Figure 3b TetR-repressor_TetR-Sin3^{C}	This work
G9E3S	G9E3 derivative; pAF9D92<br>Figure 3b TetR-repressor_TetR-Tup1	This work
G9E3T	G9E3 derivative; pAF9D93<br>Figure 3b TetR-repressor_TetR-Mig1^{C}	This work
G9E3a	G9E3 derivative; pAF9D94<br>Figure 3b TetR-repressor_TetR-Cyc8	This work
G9E3U	G9E3 derivative; pAF9C3	This work
G3A2A	CEN.PK113-5D derivative; *ura3(1, 704)::KlURA3>*T_{AgTEF1}-P_{TEF1}*>2*TcRb>yEGFP>*T_{PGK1}<br>Figure 3b: P_{TEF1}+2*TcRb, TetR-repressor_None	This work
G3A2B	CEN.PK113-5D derivative; *ura3(1, 704)::KlURA3>*T_{AgTEF1}-P_{TEF1}*>3*TcRb>yEGFP>*T_{PGK1}<br>Figure 3b: P_{TEF1}+3*TcRb, TetR-repressor_None	This work
GB5AS	CEN.PK113-5D derivative; *ura3(1, 704):: KlURA3>*T_{AgTEF1}-P_{GAL1}*>(BamHI)yEGFP*	[6]
GB5ATetR-GAL80	GB5AS derivative; *GAL80(-111, -1)*:: P_{AgTEF1}-*KanMX4*-T_{AgTEF1}-P_{HAC1}*>TetR>TUP1>*P_{ABF1}- $P_{TEF1+[TetO]}$<br>Figure 3d	This work
G8EFA	CEN.PK113-5D derivative; *ura3(1, 704):: KlURA3>*T_{AgTEF1}-P_{TEF1}*>UBI4>RHGSGTMV>*$DHFR^{P66L}$*>yEGFP>*T_{PGK1}<br>Figure 4c: P_{TEF1}-*UBI4- RHGSGTMV-*$DHFR^{*}$-*yEGFP*-T_{PGK1}	This work
GB5J3	CEN.PK113-5D derivative; *ura3(1, 704):: KlURA3>*T_{AgTEF1}-P_{GAL1}*>yEGFP*<br>*Gal80::LoxP*-P_{AgTEF1}-*KanMX4*-T_{AgTEF1}-*LoxP*	[9]
GJ3B5HdMIG1	GB5J3 derivative;<br>MIG1(-116,-1):: P_{AgTEF1}-*ble*-T_{AgTEF1}-P_{TEF2}- *UBI4- RHGSGTMV-*$DHFR^{*}$<br>Figure 4d: P_{TEF2}-*H.Degron-MIG1*	This work
GJ3B5HAC1Hd	GB5J3 derivative;<br>MIG1(-116,-1):: P_{AgTEF1}-*ble*-T_{AgTEF1}-P_{HAC1}- *UBI4- RHGSGTMV-*$DHFR^{*}$<br>Figure 4d: P_{HAC1}-*H.Degron-MIG1*	This work
GJ3B5NRG1Hd	GB5J3 derivative;<br>MIG1(-116,-1):: P_{AgTEF1}-*ble*-T_{AgTEF1}-P_{NRG1}- *UBI4- RHGSGTMV-*$DHFR^{*}$<br>Figure 4d: P_{NRG1}-*H.Degron-MIG1*	This work

G4M2	CEN.PK2-1C derivative; *ura3(1, 704):: KlURA3>*T_{AgTEF1}-P_{SeGAL2}*>(BamHI)yEGFP*<br>Figure 2c: Promoter_*Se.GAL2*, Trans-factor_None	This work
G4Q21	CEN.PK2-1C derivative; *ura3(1, 704):: KlURA3>*T_{AgTEF1}-P_{SkGAL2}*>(BamHI)yEGFP*<br>Figure 2c: Promoter_*Sk.GAL2*, Trans-factor_None	This work
G4Q22	CEN.PK2-1C derivative; *ura3(1, 704):: KlURA3>*T_{AgTEF1}-P_{SkGAL2}*M1>(BamHI)yEGFP*<br>Figure 2c: Promoter_*Sk.GAL2* (1×PZ4), Trans-factor_None	This work
G4Q23	CEN.PK2-1C derivative; *ura3(1, 704):: KlURA3>*T_{AgTEF1}-P_{SkGAL2}*M2>(BamHI)yEGFP*<br>Figure 2c: Promoter_*Sk.GAL2* (2×PZ4), Trans-factor_None	This work
G4Q25B	CEN.PK2-1C derivative; *ura3(1, 704):: KlURA3>*T_{AgTEF1}-P_{SkGAL2}*M3>(BamHI)yEGFP*<br>Figure 2c: Promoter_*Sk.GAL2* (2’×PZ4), Trans-factor_None	This work
G4Q24	CEN.PK2-1C derivative; *ura3(1, 704):: KlURA3>*T_{AgTEF1}-P_{SkGAL2}*M4>(BamHI)yEGFP*<br>Figure 2c: Promoter_*Sk.GAL2* (4×PZ4), Trans-factor_None	This work
G4Q25A	CEN.PK2-1C derivative; *ura3(1, 704):: KlURA3>*T_{AgTEF1}-P_{SkGAL2}*M5>(BamHI)yEGFP*<br>Figure 2c: Promoter_*Sk.GAL2* (5×PZ4), Trans-factor_None	This work
G4Q26	CEN.PK2-1C derivative; *ura3(1, 704):: KlURA3>*T_{AgTEF1}-P_{SkGAL2}*M6>(BamHI)yEGFP*<br>Figure 2c: Promoter_*Sk.GAL2* (1×PZ4 4×Z268), Trans-factor_None	This work
G4M2C	G4M2 derivative; pAF4EH4R<br>Figure 2c: Promoter_*Se.GAL2*, Trans-factor_Zif268-VP16[A]	This work
G4Q21C	G4Q21 derivative; pAF4EH4R<br>Figure 2c: Promoter_*Sk.GAL2* (1×PZ4), Trans-factor_Zif268-VP16[A]	This work
G4Q22C	G4Q22 derivative; pAF4EH4R<br>Figure 2c: Promoter_*Sk.GAL2* (2×PZ4), Trans-factor_Zif268-VP16[A]	This work
G4Q23C	G4Q23 derivative; pAF4EH4R<br>Figure 2c: Promoter_*Sk.GAL2* (3×PZ4), Trans-factor_Zif268-VP16[A]	This work
G4Q25BC	G4Q25B derivative; pAF4EH4R<br>Figure 2c: Promoter_*Sk.GAL2* (2’×PZ4), Trans-factor_Zif268-VP16[A]	This work
G4Q24C	G4Q24 derivative; pAF4EH4R<br>Figure 2c: Promoter_*Sk.GAL2* (4×PZ4), Trans-factor_Zif268-VP16[A]	This work
G4Q25AC	G4Q25A derivative; pAF4EH4R<br>Figure 2c: Promoter_*Sk.GAL2* (4×PZ4), Trans-factor_Zif268-VP16[A]	This work
G4Q26C	G4Q26 derivative; pAF4EH4R<br>Figure 2c: Promoter_*Sk.GAL2* (1×PZ4 4×Z268), Trans-factor_Zif268-VP16[A]	This work
G4M2N	G4M2 derivative; pAFZifPZ431<br>Figure 2c: Promoter_*Se.GAL2*, Trans-factor_ZifPZ43-Med3	This work
G4Q21N	G4Q21 derivative; pAFZifPZ431<br>Figure 2c: Promoter_*Sk.GAL2* (1×PZ4), Trans-factor_ ZifPZ43-Med3	This work
G4Q22N	G4Q22 derivative; pAFZifPZ431<br>Figure 2c: Promoter_*Sk.GAL2* (2×PZ4), Trans-factor_ ZifPZ43-Med3	This work
G4Q23N	G4Q23 derivative; pAFZifPZ431<br>Figure 2c: Promoter_*Sk.GAL2* (3×PZ4), Trans-factor_ ZifPZ43-Med3	This work

G4Q25BN	G4Q25B derivative; pAFZifPZ431<br>Figure 2c: Promoter_*Sk.GAL2* (2'×PZ4), Trans-factor_ ZifPZ43-Med3	This work
G4Q24N	G4Q24 derivative; pAFZifPZ431<br>Figure 2c: Promoter_*Sk.GAL2* (4×PZ4), Trans-factor_ ZifPZ43-Med3	This work
G4Q25AN	G4Q25A derivative; pAFZifPZ431<br>Figure 2c: Promoter_*Sk.GAL2* (4×PZ4), Trans-factor_ ZifPZ43-Med3	This work
G4Q26E	G4Q26 derivative; pAFZif7C1<br>Figure 2c: Promoter_*Sk.GAL2* (1×PZ4 4×Z268), Trans-factor_ Zif268-Med3	This work
G4M2O	G4M2 derivative; pAFZifPZ432<br>Figure 2c: Promoter_*Se.GAL2*, Trans-factor_ZifPZ43-Med15	This work
G4Q21O	G4Q21 derivative; pAFZifPZ432<br>Figure 2c: Promoter_*Sk.GAL2* (1×PZ4), Trans-factor_ ZifPZ43-Med15	This work
G4Q22O	G4Q22 derivative; pAFZifPZ432<br>Figure 2c: Promoter_*Sk.GAL2* (2×PZ4), Trans-factor_ ZifPZ43-Med15	This work
G4Q23O	G4Q23 derivative; pAFZifPZ432<br>Figure 2c: Promoter_*Sk.GAL2* (3×PZ4), Trans-factor_ ZifPZ43-Med15	This work
G4Q25BO	G4Q25B derivative; pAFZifPZ432<br>Figure 2c: Promoter_*Sk.GAL2* (2'×PZ4), Trans-factor_ ZifPZ43-Med15	This work
G4Q24O	G4Q24 derivative; pAFZifPZ432<br>Figure 2c: Promoter_*Sk.GAL2* (4×PZ4), Trans-factor_ ZifPZ43-Med15	This work
G4Q25AO	G4Q25A derivative; pAFZifPZ432<br>Figure 2c: Promoter_*Sk.GAL2* (4×PZ4), Trans-factor_ ZifPZ43-Med15	This work
G4Q26F	G4Q26 derivative; pAF7E1<br>Figure 2c: Promoter_*Sk.GAL2* (1×PZ4 4×Z268), Trans-factor_ Zif263-Med15	This work
o501R	CEN.PK2-1C derivative;<br>HMG2^{K6R}(-152,-1)::HIS3-T_{EFM1}<Ef.mvaS<P_{GAL1}-P_{GAL10}>ACS2>T_{ACS2}-P_{GAL2}> Ef.mvaE >T_{EBS1}-P_{GAL7}<br>pdc5(-31,94)::P_{GAL2}> ERG12>T_{NAT5}-P_{TEF2}>ERG8>T_{IDP1}-T_{PRM9}<MVD1<P_{ADH2}-T_{RPL15A}<IDI1<P_{TEF1}-TRP1<br>ERG9(1336, 1336)::T_{URA3}- P_{GAL7}>MVD1>T_{PRM9}-P_{GAL2}>-ERG12>T_{NAT5}-T_{IDP1}<ERG8<P_{GAL10}-P_{GAL1}>IDI1>T_{RPL15A}-P_{ACS2}>SKP1-Os.TIR1	This work
o501RB	o501R derivative;<br>GAL80(-111, -1):: P_{AgTEF1}-KanMX4-T_{AgTEF1}-P_{HAC1}>TetR>TUP1>P_{ABF1}- $P_{TEF1+[TetO]}$	This work
o5HIRB	o57BRB derivative;<br>MIG1(-116, 3):: P_{AgTEF1}-ble-T_{AgTEF1}-P_{HAC1}- UBI4- RHGSGTMV-DHFR*	This work
N9R5HIRBU	o5HIRB derivative; *URA3* pJT9RFR<br>Figure 5	This work

Supplementary Table 3: List of primers and PCR fragments used in this work. P_{XXX} and T_{XXX} indicate promoter and terminator sequence of gene XXX, respectively; sequences *in italic and red* indicate sequences complimentary to the DNA template. SGD, *S. cerevisiae* genomic DNA.

#	Overlap extension PCR fragment	PCR fragment	Primer name	Sequence (5' → 3')
1		P_{GLN3} from SGD	PPGGLN3ps	AAGGGTTGCTCGAGAAAGAGCTC *CAATACGAGCAGCAAAGAAAT*
			PPGGLN3pa	TGAATAATTCTTCACCTTTAGACAT *TTGTTTGTTGGTGGGGGAA*
2		P_{TOR1} from SGD	PPGTOR1ps	AAGGGTTGCTCGAGAAAGAGCTC *ACAGCGGGATTGTTTAAGTATG*
			PPGTOR1pa	TGAATAATTCTTCACCTTTAGACAT *ATCAATGCAAACCTGCTAGC*
3		P_{DAL80} from SGD	PPGDAL80ps	AAGGGTTGCTCGAGAAAGAGCTC *CACCCTTGTTTATCTATCCTAC*
			PPGDAL80pa	TGAATAATTCTTCACCTTTAGACAT *TCTCTTATATATAATATGATATAATATAAT G*
4		P_{GCR1} from SGD	PPGGCR1ps	AAGGGTTGCTCGAGAAAGAGCTC *CGCAATCAAGAACATGATTTC*
			PPGGCR1pa	TGAATAATTCTTCACCTTTAGACAT *TTTCAATATATATCAAAGGCCAA*
5		P_{GCR2} from SGD	PPGGCR2ps	AAGGGTTGCTCGAGAAAGAGCTC *CCTTTGGATATTCTCCCAC*
			PPGGCR2pa	TGAATAATTCTTCACCTTTAGACAT *TATGTGAGTGTAACTTTTCGTC*
6		P_{SNF1} from SGD	PPGSNF1ps	AAGGGTTGCTCGAGAAAGAGCTC *TATATTTAACTCTCGATCTTGC*
			PPGSNF1pa	TGAATAATTCTTCACCTTTAGACAT *GTTGACTTTATTAAGGGAGTG*
7		P_{YPK2} from SGD	PPGYPK2ps	AAGGGTTGCTCGAGAAAGAGCTC *AACGAATGATTTACTAATGGCT*
			PPGYPK2pa	TGAATAATTCTTCACCTTTAGACAT *GTTACTATATGTATATTTGTATACG*
8		P_{ADR1} from SGD	PPGADR1ps	AAGGGTTGCTCGAGAAAGAGCTC *TTGTTTTCATGTTTTACTTCGC*
			PPGADR1pa	TGAATAATTCTTCACCTTTAGACAT *AGTAATAGAGTATGATTATTTTTTTA*
9		P_{NRG1} from SGD	PPGNRG1ps	AAGGGTTGCTCGAGAAAGAGCTC *TCTGTTTTCTAAAAGAGGAAAAAG*
			PPGNRG1pa	TGAATAATTCTTCACCTTTAGACAT *TGCCTGAAAGTTTGCGAAGG*
10		P_{MIG1} from SGD	PPGMIG1ps	AAGGGTTGCTCGAGAAAGAGCTC *TATAATGGCTTCTCAGGAAACC*
			PPGMIG1pa	TGAATAATTCTTCACCTTTAGACAT *GGCTATGGTAGTATGTCGTC*
11		P_{ROX1} from SGD	PPGROX1ps	AAGGGTTGCTCGAGAAAGAGCTC *GTTCTTACTATTATAGTGCTC*
			PPGROX1pa	TGAATAATTCTTCACCTTTAGACAT *TGTTGATTGTCTAACTGCG*
12		P_{HAP4} from SGD	PPGHAP4ps	AAGGGTTGCTCGAGAAAGAGCTC *GAGGAACACGCGTTTCGTG*

			PPGHAP4pa	TGAATAATTCTTCACCTTTAGACAT *GATTCTTTTTTTGTTTTTTTTTTGTAC*
13		P_{HAC1} from SGD	PPGHAC1ps	AAGGGTTGCTCGAGAAAGAGCTC *TACAATGAAAAACAACACCAAG*
			PPGHAC1pa	TGAATAATTCTTCACCTTTAGACAT *AGTGGCGGTTGTTGTCGTAG*
14		P_{UPC2} from SGD	PPGUPC2ps	AAGGGTTGCTCGAGAAAGAGCTC *GGCAGTTGATAAGCGCTTC*
			PPGUPC2pa	TGAATAATTCTTCACCTTTAGACAT *ACTGCTGAACTGTAAATATTTG*
15	P_{TEF1+}	P_{TEF1} UAS from SGD	PPGTEF1ps2	AAGGGTTGCTCGAGAAAGAGCTC *CAGAAAGCGACCACCCAAC*
			PPGTEF1M1a	CGAGGATTGCCGGCCCGGGAATCTTA GATTCAGGTAG*AACGTGATAAAAATTTT ATTGC*
		P_{TEF1} core from SGD	PPGTEF1M1s	TAAGATTCCCGGGCCGGCAATCCTCGA GCAGAT*TCTTTTTCTTGAAAATTTTTTTTT TTG*
			PPGTEF1pa4	CAGTGAATAATTCTTCACCTTTAGA CAT TTTGGATCC*TTTGTAATTAAAACTTAGAT TAG*
16		$P_{CYC1+MalO}$ from gBlock	PPGP15MALps	AAGGGTTGCTCGAGAAAGAGCTC *AAGCACGCGGGGATAATG*
			PPGP15MALpa	TGAATAATTCTTCACCTTTAGACAT TTTGGATCC *GTTTGTGTGTCTATAGAAGTATAG*
17		P_{CYC1} UAS + from gBlock	PPGP15Cores	GGGTTGCTCGAGAAAGAGCTCACGGC GGCCAAGCACGCG
			PPGP15Corea	ATCTGCTCGAGGATTGCCGGCCCGGG AATC
18		Z268 elements + P_{TEF1} core from pILGFP2BC9	PZ3elements	CTACCTGAATCTAAGATTGGGCCCGCG TGGGCGAATTGGTGCGTGGGCGTAAG CGGGGGCGGGCGTGGGCGTAGCGTGG GCG*GCCGGCAATCCTCGAGCA*
			PPGTEF1pa4	As above
19		Z268 elements + P_{CYC1} core from pILGFP3E9	PZ3elements	As above
			PPGP15MALpa	As above
20	P_{HAC1}-Zif268-VP16-P_{ABF1}	P_{HAC1} core from SGD	PMDHAC1ps	CACTAAAGGGAACAAAAGCTG GAGCTC *TACAATGAAAAACAACACCAAG*
			PMDHAC1pa	ACAAGCGTATGGTCTAGTACCCATGAT GGATCC *AGTGGCGGTTGTTGTCGTAG*
		Zif268-VP16 (gBlock)		
		P_{ABF1} core from SGD	PMDABF1ts	GGTATTGATGAATACGGTGGTTAACCT GCAGG*ATACCCAGTTGAGAAGACG*
			PMDABF1ta	CTA TAG GGC GAA TTG GGT ACCGGGGCCC*GATATACATGTGATTAAT ATCAGAAG*

21	Fragment #5GA1	Fragment#5GC from pAF4E4H	PAFTRP1s	*GGTGACTATTGAGCACGTGAGTATACGTGATTAAG*
			PAFTRP1ma	*GTCAGCATCGGAATC*CAA*AGCACATTCTGCGGCCTC*
		Fragment #6A1 from pAF4E4H	PAFTRP1ms	*GAGGCCGCAGAATGTGCT*TTG*GATTCCGATGCTGAC*
			PAFdKpnIa	TGATATTAATCACATGTATATCGGGCCC *CAATTCGCCCTATAGTGAGTC*
22		10*WD from pAF4E4H	PAF10WDs	GTTGGTACCTGGGATTGGGATTGGGACTGGGATTGGGACTGGGACTGGGATTGGGATTGGGACTGGGACTAACCTGCAGG*ATACCCAGTTGAGAAGACG*
			PMDABF1ta	As above
23		*MED3* from SGD	PAFMED3s1	CTAAGAAGAAAAGAAAGGTTGGTACC *GACTCGATTATACCGGCAG*
			PAFMED3a1	CTTCTCAACTGGGTATCCTGCAGGTTA *CAAGAAATCCATGTTCAGACC*
24		*MED15* from SGD	PAFMED15s1	CTAAGAAGAAAAGAAAGGTTGGTACC *TCTGCTGCTCCTGTCCAAG*
			PAFMED15a1	CTTCTCAACTGGGTATCCTGCAGGTTA *AGTAGCACTTGTCCAATTATTC*
25		*GCN4*^{A} from SGD	PAFGCN4ADs1	CTAAGAAGAAAAGAAAGGTTGGTACC *ACTCCAATGTTTGAGTATGAAAAC*
			PAFGCN4ADa1	CTTCTCAACTGGGTATCCTGCAGGTTA *GGATTCAATTGCCTTATCAG*
26		*MIG1*^{C} from SGD	PAFMIG1Cs1	CTAAGAAGAAAAGAAAGGTTGGTACC *TTAAATACGGCAAATTTTTCACC*
			PAFMIG1Ca1	CTTCTCAACTGGGTATCCTGCAGGTTA *GTCCATGTGTGGGAAGGGCAAC*
27		*SIN3*^{C} from SGD	PAFSIN3Cs1	CTAAGAAGAAAAGAAAGGTTGGTACC *AGAACGTCTCTCACTGAACA*
			PAFSIN3Ca1	CTTCTCAACTGGGTATCCTGCAGGTTA *TTGAATCTTAGCCCCCTTGTC*
28		*TUP1* from SGD	PAFTUP1s1	CTAAGAAGAAAAGAAAGGTTGGTACC *ACTGCCAGCGTTTCGAATAC*
			PAFTUP1a1	CTTCTCAACTGGGTATCCTGCAGGTTA *ATTTGGCGCTATTTTTTTATACTTCC*
29	P_{HAC1}-*LacI*	P_{HAC1} core from SGD (as above) *LacI* gBlock	PMDHAC1ps	CACTAAAGGGAACAAAAGCTGGAGCTC *TACAATGAAAAACAACACCAAG*
			PAFLacIa	GTATTCGAAACGCTGGCAGTGCTAGC*AACCTTTCTTTTCTTCTTAGG*
30		*TetR* from gBlock	PAFTetRs	CGACAACAACCGCCACTCCGCGGATC *ATGTCCAGATTGGATAAGTC*
			PAFTetRa1	TGTTCAGTGAGAGACGTTCT *GCTAGCAACCTTTCTTTTCT*
31	*TetR-CYC8*	*TetR* from gBlock (as above)	PAFTetRs	As above
			PAFTetRa1	As above

		CYC8 from SGD	PAFCYC8s	CTAAGAAGAAAAGAAAGGTTGCTAGC *AATCCGGGCGGTGAACAAAC*
			PAFCYC8a	CTTCTCAACTGGGTATCCTGCAGGTTA *GTCGTCGTAGTTTTCATCTTC*
32	Tetracycline riboswitches		PTetRibos	*CATAGCAATCTAATCTAAGTTTTAATTACAAAC*
		Tetracycline Riboswitch 1	PTetRRibo1s	CATAGCAATCTAATCTAAGTTTTAATTACAAACGGCC*TAAAACATACCAGATCGCCAC*
			PTetRRiboTem	TAAAACATACCAGATCGCCACCCGCGCTTTAATCTGGAGAGGTGAAGAATACGACCACCT
			PTetRRibo1a	TTGAATTGAGAGTTGTTGATTTGCGAGTTGGCCT*AGGTGGTCGTATTCTTCAC*
		Tetracycline Riboswitch 2	PTetRRibo2s	AACTCGCAAATCAACAACTCTCAATTCAAGGCC*TAAAACATACCAGATCGCCAC*
			PTetRRiboTem	As above
			PTetRRibo2a	TCGAGTTGTTGTTGTTGTTTTGTTGTTGAGGCCT*AGGTGGTCGTATTCTTCAC*
		Tetracycline Riboswitch 2	PTetRRibo3s	TCAACAACAAAACAACAACAACAACTCGAGGCC*TAAAACATACCAGATCGCCAC*
			PTetRRiboTem	As above
			PTetRRibo3a	CACCAGTGAATAATTCTTCACCTTTAGACATTTTGGCCT*AGGTGGTCGTATTCTTCAC*
			PTetRiboa	*CACCAGTGAATAATTCTTCACCTTTAGACATTTT*
33	*ZifPZ4-MED3*		PAFZIF64s	*CGACAACAACCGCCACTCCGCGGATCATGTTGGAACCAGGTGAAAAACC*
		ZifPZ4 gBlock		
		MED3 (2) from SGD	PAFMED3s	GTTGGTACC *GACTCGATTATACCGGCAG*
			PAFMED3a	TATCCTGCAGGTTA *CAAGAAATCCATGTTCAGACC*
34		ZifPZ44 from pAFZifPZ421	PAFZifPZ44s	CAACCGCCACTCCGCGGATCATGGGTACTAGACCATACGCT *TACAAGTGTAAGCAATGTG*
			PAFZIF64a	CTGCCGGTATAATCGAGTC *GCTAGCAACCTTTCTTTTCTTC*
35	$P_{TEF1+[AguO]}$	$P_{TEF1+[AguO]}$ part 1 from SGD	PPGTEF1ps2	As above
			PIRPcore-AguOaada	GATAAAAATCGGACACCAATTATCCGATAAAAATCGGAC*TAGGGCCCAATCTTAGATTC*
			PIRPcore-AguOa	ACCCGCCCCCGCTTAATCC*GATAAAAATCGGACACCAATT*
		$P_{TEF1+[AguO]}$ part 2 from SGD	PIRPcore-AguOs	GGATTAAGCGGGGGCGGGTCC*GATTTTTATCGGATGGGCTTA*
			PIRPcore-AguOsada	GATTTTTATCGGATGGGCTTAGTCCGATTTTTATCGGAT*GCCGGCAATCCTCGAGCA*

			PPGTEF1pa4	As above
36	$P_{TEF1+[SmeO]}$	$P_{TEF1+[SmeO]}$ part 1 from SGD	PPGTEF1ps2	As above
			PIRPcore-SmeOa	CCGCCCCCGCTTATATACATACATGCTTGTTTGTTTGTAAA*TAGGGCCCAATCTTAGATT*
		$P_{TEF1+[SmeO]}$ part 2 from SGD	PIRPcore-SmeOs	ATATAAGCGGGGGCGGTTTACAAACAAACAAGCATGTATGTATA*GCCGGCAATCCTCGAG*
			PPGTEF1pa4	As above
37	$P_{TEF1+[LmrO]}$	$P_{TEF1+[LmrO]}$ part 1 from SGD	PPGTEF1ps2	As above
			PIRPcore-LmrOaada	GTGACTGGTCTATTATCTTGATT*TAGGGCCCAATCTTAGATTC*
			PIRPcore-LmrOa	AACCGCCCCCGCTTAAATCAAAAATATA*GTGACTGGTCTATTATCTTG*
		$P_{TEF1+[LmrO]}$ part 2 from SGD	PIRPcore-LmrOs	TTTAAGCGGGGGCGGTTCCTCCAATTTA*GACCAGTCTATATATTATCATTC*
			PIRPcore-LmrOsada	GACCAGTCTATATATTATCATTC*GCCGGCAATCCTCGAGCA*
			PPGTEF1pa4	As above
38	$P_{TEF1+[CymO]}$	$P_{TEF1+[CymO]}$ part 1 from SGD	PPGTEF1ps2	As above
			PIRPcore-CymOaada	TTTGTTACCAATTATAATACAGACAGGTTGGTTTGTTTCTT*TAGGGCCCAATCTTAGATT*
			PIRPcore-CymOa	TCCGCCCCCGCTTATACAAACAGACCAGATTGTCTG*TTTGTTACCAATTATAATACAGA*
		$P_{TEF1+[CymO]}$ part 2 from SGD	PIRPcore-CymOs	TATAAGCGGGGGCGGAAGAAACAAACCAACCTGTCTGTATTAT*GCCGGCAATCCTCGAGC*
			PPGTEF1pa4	As above
39	$P_{TEF1+[PhlO]}$	$P_{TEF1+[PhlO]}$ part 1 from SGD	PPGTEF1ps2	As above
			PIRPcore-PhlOaada	CCTTAACGATACGGTACGTTTCGTATCATACATA*TAGGGCCCAATCTTAGATTC*
			PIRPcore-PhlOa	TACCGCCCCCGCTTAACGCTA*CCTTAACGATACGGTACG*
		$P_{TEF1+[PhlO]}$ part 2 from SGD	PIRPcore-PhlOs	GTTAAGCGGGGGCGGTATGTATGATA*CGAAACGTACCGTATCGTT*
			PIRPcore-PhlOsa	CGAAACGTACCGTATCGTTAAGGTAGCGT*GCCGGCAATCCTCGAGCA*
			PPGTEF1pa4	As above

40	$P_{TEF1+[CamO]}$	$P_{TEF1+[CamO]}$ part 1 from SGD	PPGTEF1ps2	As above
			PIRPcore-CamOaada	AAGCCCACCACGCATGCTCAGTATATCGCAGATATAGAGCCTG*TAGGGCCCAATCTTAGA*
			PIRPcore-CamOa	GCCGCCCCCGCTTATGCTCAGTATATCGCAGATATAGAGCCTG*TAAGCCCACCACGCATG*
		$P_{TEF1+[CamO]}$ part 2 from SGD	PIRPCore-CamOs	AAGCGGGGGCGGCAGGCTCTATATCTGCGATATACTGAGCAT*GCCGGCAATCCTCGAGCA*
			PPGTEF1pa4	As above
41	$P_{TEF1+[CelO]}$	$P_{TEF1+[CelO]}$ part 1 from SGD	PPGTEF1ps2	As above
			PIRPcore-CelOaada	AGCGCTCCCAACCAATTTGGGAGCGCTCCCA*TAGGGCCCAATCTTAGATTC*
			PIRPcore-CelOa	CACCGCCCCCGCTTATGGGAGCGCTCCCATAAGCCCTGGG*AGCGCTCCCAACCAATTT*
		$P_{TEF1+[CelO]}$ part 2 from SGD	PIRPcore-CelOs	CATAAGCGGGGGCGGTGGGAGCGCTCCCA*GCCGGCAATCCTCGAGCA*
			PPGTEF1pa4	As above
42	$P_{TEF1+[MalO]}$	$P_{TEF1+[MalO]}$ part 1 from SGD	PPGTEF1ps2	As above
			PIRPcore-MalOa	ACCGCCCCCGCTTACCTCATCCCCACCAATTCCTCCTCCCC*TAGGGCCCAATCTTAGATT*
		$P_{TEF1+[MalO]}$ part 1 from SGD	PIRPcore-MalOs	GTAAGCGGGGGCGGTAGGGGAGGAGGAGCAGGGGATGAGGTA*GCCGGCAATCCTCGAGC*
			PPGTEF1pa4	As above
42	$P_{TEF1+[LexO]}$	$P_{TEF1+[LexO]}$ part 1 from SGD	PPGTEF1ps2	As above
			PIRPcore-LexOaada	TATATATACAGTAACCAATTTACTGTATGATCATACAGTA*TAGGGCCCAATCTTAGATTC*
			PIRPcore-LexOa	ACCGCCCCCGCTTATACTGTA*TATATATACAGTAACCAATTTAC*
		$P_{TEF1+[LexO]}$ part 2 from SGD	PIRPCore-LexOs	ATAAGCGGGGGCGGTACTGTATGCGCATACAGTAG*GGCTTATACTGTGTCACCTG*
			PIRPCore-LexOsada	GGCTTATACTGTGTCACCTGACAGTA*GCCGGCAATCCTCGAGCA*
			PPGTEF1pa4	As above
43	P_{HAC1}-*TetR*-*TUP1*-T_{ABF1}-$P_{CYC1+[TetO]}$	*PHAC1-TetR* from pAF9D92	P3DH8HAC1padas	GAACAATAAGACAGGACTGTAAAGTCTTCACTATAAGAAA*ATCACACGAGCGCCCGGACG*

			P3DH8HAC1ps	ATCACACGAGCGCCCGGACGATGTCTCTGTTTAATTAA*TACAATGAAAAACAACACCAAG*
			P3DH8TetRa	CAACTTTCTCTTCTTTCTTTTAGCAGTTGTAGAGGTAGA*GGAGCCAGATTCACATTTCAA*
		$TUP1$-T_{ABF1} from pAF9D92	P3DH8TUP1s	TCTACCTCTACAACTGCTAAAAGAAAGAAGAGAAAGTTG*ACTGCCAGCGTTTCGAATAC*
			P3DH8ABF1ta	CCCAATCTTAGATTCAGGTAGGCGGCCGC*GATATACATGTGATTAATATCAGAAG*
		TetO from pILGFP9E3	P3DH8TetOs	CTTCTGATATTAATCACATGTATATCGCGGCCGC*CTACCTGAATCTAAGATTGGG*
			P3DH8TetOa	TATATACACGCCTGGCGGATC*TGCTCGAGGATTGCCGGCAC*
		P_{CYC1} from SGD	P3DH8CYC1PCores	GTGCCGGCAATCCTCGAGCA*GATCCGCCAGGCGTGTATATA*
			P3DH8CYC1PCorea	CGAAGATCTCTTGTTGTAGTCCATTTTACTAGT*TTTGGATCCGTTTGTGTGTC*
44	*ARM_{GAL80U}-KanMX4*	*ARM_{GAL80U}* from SGD	PIRGAL80us1	GTAAAACGACGGCCAGTGAATTCGTTT*AAACCAACCTAAAGGTATTAACTTC*
			PIRGAL80ua1	TGATGTATATATATTCAGGTTCATTACCCTGTT*ATGAGCTTTTGGTTATTATGAATGT*
		KanMX4-part 1 from pUG6	PIRKanMX4s	GTAATGAACCTGAATATATATACATCATTAATTCCATGG*GTTTAGCTTGCCTCGTCCC*
			PIRKanMX4ina	*CTGAGCGAGACGAAATACGCGATCGC*
		KanMX4-part 2 from pUG6	PIRKanMX4ins	*CATGGCAAAGGTAGCGTTGCCAATGATG*
			PIRKanMX4adaa	TGATGTATATATATTCAGGTTCATTACCCTGTTATCCCTA*GTTTTCGACACTGGATGGCG*
			PIRKanMX4adaa1	GCTTTTGGCAGCTTGGTGTTGTTTTTCATTGTA TTAATTAA*TGATGTATATATATTCAGG*
			PIRKanMX4a1	*GCTTTTGGCAGCTTGGTGTTGTTTTTCATTGTA*
45		*$P_{TEF1+[TetO]}$* from pILGFP9E3	PIRGALTEF1ps	CTGATATTAATCACATGTATATCGCGGCCGC*CAGAAAGCGACCACCCAAC*
			PIRGALTEF1pa	GATCTCTTGTTGTAGTCCATTTTACTAGT*TTTGTAATTAAAACTTAGATTAGATTG*
46	*UBI4-R-DHFR**	*UBI4* from SGD	PPGUBIs	TAGCAATCTAATCTAAGTTTAATTACAAA *ATGCAGATTTTCGTCAAGACT*
			PPGUBIa	GAAACAGCAACGATACAGTTCAATGGTCTTCT*ACCACCTCTTAGCCTTAGCA*
		*DHFR** from gBlock		
			PPGDFHRa	CAGTGAATAATTCTTCACCTTTAGAACCACCACC*ACCACCACCATCCTTCTTTTCGTAGACTTC*

47	*UBI4-RHGSGTMV-DHFR**	*UBI4* from SGD	PPGUBIs	As above
			PPGUBIa1	GGTCTAACCATGATACCAGAACCATGTCT *ACCACCTCTTAGCCTTAGCA*
		*DHFR** from pILGFP2DA	PPGDFHRs	GGTGGT AGA CAT GGT TCT GGT ATC ATG GTT*AGACCATTGAACTGTATCGT*
			PPGDFHRa	As above
48	$P_{Sk.GAL2}$ M1	$P_{Sk.GAL2}$ M1 part 1 from pILGFP4Q	PPGPromoters	*CATACATTATACGAAGTTATATTAAGGGTTGCTCGAGAAA*
			PFPPSkGAL2M1a	ACGACTGCTAACAACGTCTCACTCTAAAAACGTCTCACTCTA*GTTTAGCTTTACAGGAGG*
		$P_{Sk.GAL2}$ M1 part 2 from pILGFP4Q	PFPPSkGAL2M1s	CCTCCTGTAAAGCTAAACTAGAGTGAGACGT*TTTTAGAGTGAGACGTTGTTAGCAGTCGT*
			PPGPromotera	*GGGACAACACCAGTGAATAATTCTTCACCTTTAGA*
49	$P_{Sk.GAL2}$ M2	$P_{Sk.GAL2}$ M2 part 1 from pILGFP4Q	PFPPSkGAL2ps	As above
			PFPPSkGAL2M1a	As above
		$P_{Sk.GAL2}$ M2 part 2 from pILGFP4Q	PFPPSkGAL2M2s	AACTAGAGTGAGACGTTTTTAGAGTGAGACGTTGTTAGAGTGAGA*CGTTGTTCAAGGGAG*
			PFPPSkGAL2pa	As above
50	$P_{Sk.GAL2+[PZ4]}$	$P_{Sk.GAL2}$ part 1 from pILGFP4Q	PFPPSkGAL2ps	As above
			PPGSkGAL2m3a	CCAATCTTAGATTCAGGTAG*ATAATTGACTTTTGTTCCTATTTC*
		[PZ4] elements from pILGFP8H8	PPGSkGAL2motifs	GAAATAGGAACAAAAGTCAATTAT*CTACCTGAATCTAAGATTGG*
			PPGSkGAL2motifa	GTTTAGCTTTACAGGAGGAG*CTGCTCGAGGATTGCCG*
		$P_{Sk.GAL2}$ part 1 from pILGFP4Q	PPGSkGAL2m3s	CGGCAATCCTCGAGCA*GCTCCTCCTGTAAAGCTAAAC*
			PFPPSkGAL2pa	As above
51	$P_{Sk.GAL2+[Z268]}$	$P_{Sk.GAL2}$ part 1 from pILGFP4Q	PFPPSkGAL2ps	As above
			PPGSkGAL2m3a	As above
		[Z268] elements from pILGFP4D41	PPGSkGAL2motifs	As above
			PPGSkGAL2motifa	As above

		$P_{Sk.GAL2}$ part 1 from pILGFP4Q	PPGSkGAL2m3s	As above
			PFPPSkGAL2pa	As above
52		*ble* from pUG66	PHDMIG1bleS	TTCTTGGATAATTTATTTATTATAACCCTTTTTTTTCACC*CCAGTACTCATTAACGAAGA*
			PHDMIG1bleSadaR1	CCAGTACTCATTAACGAAGAACTTTGTTATGTAGAGT*TTTTTAGCTACCTATATTCCAC*
			PHDMIG1bleSada1R2	TTTTTAGCTACCTATATTCCACTAGGGATAACAGGGTAAT*CAGCTGAAGCTTCGTACGC*
			PHDMIG1blea	*GCATAGGCCACTAGTGGATCTGATATCACCTA*
53	P_{TEF2}-*H.Degron*	P_{TEF2} from SGD	PHDMIG1TEF2ps	GAAGTTATTAGGTGATATCAGATCCACTAGTGGCCTATGC*ACTTTGTTATGTAGAGTTTT*
			PHDMIG1TEF2pa	TTATGGTTTTACCGGTCAAAGTCTTGACGAAAATCTG*CATGTTTAGTTAATTATAGTTCG*
		H.Degron from pILGFP8EFA	PHDMIG1Hdegrons	CCTTCGCAAACTTTCAGGCA*ATGCAGATTTTCGTCAAGACTTTG*
			PHDMIG1Hdegrona1	CATCAACGTTAGACACTTGTGTCATTGGATATGGGCTTTG*ACCACCACCACCACCACCAT*
54		*ble* from pUG66	PHDMIG1bleS3	TTATAACCCTTTTTTTTCACCCCAGTACTCATTAACGAAGA *CAGCTGAAGCTTCGTACGC*
			PHDMIG1blea	As above
55	P_{HAC1}-*H.Degron*	P_{HAC1} from SGD	PHDMIG1HAC1ps1	GAAGTTATTAGGTGATATCAGATCCACTAGTGGCCTATGC *AATGAAAAACAACACCAAGC*
			PHDMIG1HAC1pa1	TTATGGTTTTACCGGTCAAAGTCTTGACGAAAATCTG *CAT AGTGGCGGTTGTTGTCGTAG*
		H.Degron from pILGFP8EFA	PHDMIG1Hdegrons	As above
			PHDMIG1Hdegrona1	As above
56	P_{NRG1}-*H.Degron*	P_{NRG1} from SGD	PHDMIG1NRG1ps1	GAAGTTATTAGGTGATATCAGATCCACTAGTGGCCTATGC*TTCCGCATATTTGTCTTTGA*
			PHDMIG1NRG1pa1	TTATGGTTTTACCGGTCAAAGTCTTGACGAAAATCTG*CATTGCCTGAAAGTTTGCGAAGG*
		H.Degron from pILGFP8EFA	PHDMIG1Hdegrons	As above
			PHDMIG1Hdegrona1	As above

57	*URA3* from SGD	PPGURA3Fs	ATGTGGCTGTGGTTTCAGGGTC
		PPGURA3Fa	TTCAATGCGTCCATCTTTACAGTCCT

Supplementary Table 4. Construction of the plasmids used in this work. Numbers refer to DNA fragments listed in Supplementary Table 3.

Plasmid	Construction process
pILGFP1E8	**Fragment P_{GLN3} (#1) was cloned into *Bam*HI site of pILGFP1D5 through Gibson assembly to generate pILGFP1E8, and:**
pILGFP1G8	Fragment P_{TOR1} (#2) to generate pILGFP1G8
pILGFP1A9	Fragment P_{DAL80} (#3) to generate pILGFP1A9
pILGFP1C9	Fragment P_{GCR1} (#4) to generate pILGFP1C9
pILGFP1E9	Fragment P_{GCR2} (#5) to generate pILGFP1E9
pILGFP1G9	Fragment P_{SNF1} (#6) to generate pILGFP1G9
pILGFP1AA	Fragment P_{YPK2} (#7) to generate pILGFP1AA
pILGFP1CA	Fragment P_{ADR1} (#8) to generate pILGFP1CA
pILGFP1EA	Fragment P_{NRG1} (#9) to generate pILGFP1EA
pILGFP1GA	Fragment P_{MIG1} (#10) to generate pILGFP1GA
pILGFP1CB	Fragment P_{ROX1} (#11) to generate pILGFP1CB
pILGFP1EB	Fragment P_{HAP4} (#12) to generate pILGFP1EB
pILGFP1GB	Fragment P_{HAC1} (#13) to generate pILGFP1GB
pILGFP4D5	Fragment P_{UPC2} (#14) to generate pILGFP4D5
pILGFP2BC9	Fragment P_{TEF1+} (#15) to generate pILGFP2BC9
pILGFP2H8	Fragment $P_{CYC1+MalO}$ (#16) to generate pILGFP2H8
pILGFPTEF1-AguO	Fragment $P_{TEF1+[AguO]}$ (#35) to generate pILGFPTEF1-AguO
pILGFPTEF1-SmeO	Fragment $P_{TEF1+[SmeO]}$ (#36) to generate pILGFPTEF1-SmeO
pILGFPTEF1-SmeO	Fragment $P_{TEF1+[LmrO]}$ (#37) to generate pILGFPTEF1-SmeO
pILGFPTEF1-CymO	Fragment $P_{TEF1+[CymO]}$ (#38) to generate pILGFPTEF1-CymO
pILGFPTEF1-PhlO	Fragment $P_{TEF1+[PhlO]}$ (#39) to generate pILGFPTEF1-PhlO
pILGFPTEF1-CamO	Fragment $P_{TEF1+[CamO]}$ (#40) to generate pILGFPTEF1-CamO
pILGFPTEF1-CelO	Fragment $P_{TEF1+[CelO]}$ (#41) to generate pILGFPTEF1-CelO
pILGFPTEF1-MalO	Fragment $P_{TEF1+[MalO]}$ (#42) to generate pILGFPTEF1-MalO
pILGFPTEF1-LexO	Fragment $P_{TEF1+[LexO]}$ (#43) to generate pILGFPTEF1-LexO
pILGFP10E1A	**Fragment** $P_{Sk.GAL2}$ M1 **(#48) was cloned into *Bam*HI site of pILGFP3 through Gibson assembly to generate pILGFP10E1A, and:**
pILGFP10E1B	Fragment $P_{Sk.GAL2}$ M2 (#49) to generate pILGFP10E1B
pILGFP10CF72/pILGFP10CF72 (B9)/pILGFPCF72 (B11)	Fragment $P_{Sk.GAL2+[PZ4]}$ (#50) to generate pILGFP10CF72/pILGFP10CF72 (B9)/pILGFPCF72 (B11). Sanger Sequencing was used to verify the sequences of resulting plasmids, showing that these three plasmids have a variety number of [PZ4] elements.
pILGFP10CF71	Fragment $P_{Sk.GAL2+[Z268]}$ (#51) to generate pILGFP10CF71
pILGFP3E9	Fragment P_{CYC1} UAS + (#17) was cloned into *Nae*I/*Sac*I sites of pILGFP2H8 through Gibson Assembly to generate pILGFP3E9

pILGFP4D41	Fragment Z268 elements + P_{TEF1} core (#18) was cloned into *Nae*I/*Bam*HI sites of pILGFP2BC9 through Gibson Assembly to generate pILGFP4D41
pILGFP4D42	Fragment Z268 elements + P_{CYC1} core (#19) was cloned into *Nae*I/*Bam*HI sites of pILGFP3E9 through Gibson Assembly to generate pILGFP4D42
pILGFP8H8	A gBlock fragment containing PZ4 elements and *TEF1* core promoter was cloned into *Nae*I/*Bam*HI sites of pILGFP2BC9 through Gibson Assembly to generate pILGFP8H8
pILGFP9E3	A gBlock fragment containing TetO elements and *TEF1* core promoter was cloned into *Nae*I/*Bam*HI sites of pILGFP2BC9 through Gibson Assembly to generate pILGFP9E3
pILGFP3A2 (1) / pILGFP3A2 (2)	Fragment Tetracycline riboswitches (#32) was cloned into *Bam*HI site of pILGFP1F5 through Gibson Assembly to generate pILGFP3A2 (1) / pILGFP3A2 (2). Sanger sequencing was used to verify the sequences of resulting plasmids, showing that these three plasmids have two or three riboswitches.
pILGFP2DA	Fragment *UBI4-R-DHFR** (#46) was cloned into *Bam*HI site of pILGFP1F5 through Gibson Assembly to generate pILGFP2DA
pILGFP8EFA	Fragment *UBI4- RHGSGTMV-DHFR** (#47) was cloned into *Bam*HI site of pILGFP1F5 through Gibson Assembly to generate pILGFP8EFA
pAF4EH4	Fragment P_{HAC1}-*Zif268-VP16*-P_{ABF1} (#20) was cloned into *Sac*I/*Apa*I site of pRS414 through Gibson Assembly to generate pAF4EH4
pAF4EH4R	Fragment #5GA1 (#21) was cloned into *Bst*Z17I/*Apa*I site of pAF4EH4 through Gibson Assembly to generate pAF4EH4R
pAF5HB	*Kpn*I/*Apa*I-digested fragment 10*WD (#22) was cloned into *Kpn*I/*Apa*I sites of pAF4EH4R through ligation to generate pAF5HB
pAF7C1	**Fragment *MED3* (#23) was cloned into *Kpn*I/*Sbf*I sites of pAF5HB through Gibson Assembly to generate pAF7C1, and:**
pAF7E1	Fragment *MED15* (#24) to generate pAF7E1
pAF7G1	Fragment $GCN4^{A}$ (#25) to generate pAF7G1
pAF7E2	Fragment $MIG1^{C}$ (#26) to generate pAF7E2
pAF7A2	Fragment *TUP1* (#27) to generate pAF7A2
pAF7C2	Fragment $SIN3^{C}$ (#28) to generate pAF7C2
pAF9C3	A gBlock fragment RevTetR r1.7 was cloned into *Bam*HI/*Kpn*I sites of pAF7A2 to generate pAF9C3
pAF4E9D	Fragment P_{HAC1}-*LacI* (#29) was cloned into *Sac*I/*Kpn*I of pAF7C2 through Gibson Assembly to generate pAF4E9D
pAF9D91	Fragment TetR (#30) was cloned into *Kpn*I/*Sac*II sites of pAF9C3 to generate pAF9D91
pAF9D92	Fragment TetR (#30) was cloned into *Kpn*I/*Sac*II sites of pAF4E9D to generate pAF9D92
pAF9D93	Fragment TetR (#30) was cloned into *Bam*HI/*Sal*I sites of pAF7E2 to generate pAF9D93
pAF9D94	Fragment TetR-CYC8 (#31) was cloned into *Bam*HI/*Sbf*I sites of pAF7C1 to generate pAF9D94

pAF8A5B	Fragment *ZifPZ4-MED3* (#33) was cloned into *Bam*HI/*Sbf*I sites of pAF7C1 to generate pAF8A5B
pAFZifPZ421	**A gBlock fragment ZifPZ42 was cloned into *Sac*II/*Kpn*I sites of pAF8A5B to generate pAFZifPZ421, and:**
pAFZifPZ431	A gBlock fragment ZifPZ43 to generate pAFZifPZ431
pAFZifPZ441	A gBlock fragment ZifPZ44 to generate pAFZifPZ441
pAFTALPZ41	A gBlock fragment TALPZ4 to generate pAFZifPZ441
pAF9H6	A gBlock fragment TALPZ4 was cloned into *Bam*HI/*Kpn*I sites of pAF7E1 to generate pAF9H6
pAFZifPZ422	**A gBlock fragment ZifPZ42 was cloned into *Sac*II/*Kpn*I sites of pAF9H6 to generate pAFZifPZ422, and:**
pAFZifPZ432	A gBlock fragment ZifPZ43 to generate pAFZifPZ432
pAFZifPZ442	A gBlock fragment ZifPZ44 to generate pAFZifPZ442

Supplementary Table 5. Construction of the ILHA series strains used in this work. Plasmids refer to Supplementary Table 1. DNA fragments refer to Supplementary Table 3. Biological replicates for each G_ strain were mixed and stored as mixture in glycerol stock. Each biological replicate for each N_ strain was stored as an isolate in glycerol stock. For construction-in-process o_ strains, several clones were mixed and stored as mixture in glycerol stock after verification through proper PCR reactions.

Strain	Construction process
G1E8	**Plasmid pILGFP1E8 digested with *Swa*I was transformed into CEN.PK113-5D to generate strain G1E8, and:**
G1G8	pILGFP1G8 to generate G1G8
G1A9	pILGFP1A9 to generate G1A9
G1C9	pILGFP1C9 to generate G1C9
G1E9	pILGFP1E9 to generate G1E9
G1G9	pILGFP1G9 to generate G1G9
G1AA	pILGFP1AA to generate G1AA
G1CA	pILGFP1CA to generate G1CA
G1EA	pILGFP1EA to generate G1EA
G1GA	pILGFP1GA to generate G1GA
G1CB	pILGFP1CB to generate G1CB
G1EB	pILGFP1EB to generate G1EB
G1GB	pILGFP1GB to generate G1GB
G5E4	pILGFP5E4 to generate G5E4
G2BC9	pILGFP2BC9 to generate G2BC9
G3E9	pILGFP3E9 to generate G3E9
G2DA	pILGFP2DA to generate G2DA
G8EFA	pILGFP8EFA to generate G8EFA
G3A2A	pILGFP3A2 (1) to generate G3A2A
G3A2B	pILGFP3A2 (2) to generate G3A2B
G4D41	**Plasmid pILGFP4D41 digested with *Swa*I was transformed into CEN.PK2-1C to generate strain G4D41, and:**
G4D42	pILGFP4D42 to generate G4D42
G8H8	pILGFP8H8 to generate G8H8
G9E3	pILGFP9E3 to generate G9E3
GB5ATetR-GAL80	*Pme*I-digested pIRTetR-GAL80 was transformed into GB5AS to generate strain GB5ATetR-GAL80
GJ3B5HdMIG1	Fragments *ble* (#52) and P_{TEF2}-*H.Degron* (#53) were co-transformed into strain GJ3B5A to generate strain GJ3B5HdMIG1
GJ3B5HAC1Hd	Fragments *ble* (#54) and P_{HAC1}-*H.Degron* (#55) were co-transformed into strain GJ3B5A to generate strain GJ3B5HAC1Hd
GJ3B5NRG1Hd	Fragments *ble* (#54) and P_{NRG1}-*H.Degron* (#56) were co-transformed into strain GJ3B5A to generate strain GJ3B5NRG1Hd
o501RB	PmeI-digested pIRTetR-GAL80 was transformed into o501R to generate strain o501RB<br>Note: 125 μM tetracycline was supplemented for strain maintenance.

o5HIRB	Fragments *ble* (#54) and P_{HAC1}-*H.Degron* (#55) were co-transformed into strain o501RB to generate strain o5HIRB<br>Note: 125 µM tetracycline was supplemented for strain maintenance.
N9R5HIRBU	Step 1: Fragment URA3 (#57) was transformed into o5HIRB<br>Step 2: Plasmid pJT9RFR was transformed into step 1 strain to generate strain N9R5HIRBU<br>Note: 125 µM tetracycline was supplemented for strain maintenance.
Others (G4D41A, G4D41C, G4D41D, G4D41E, G4D41F, G4D41G, G4D41J, G4D42A, G4D42C, G4D42D, G4D42E, G4D42F, G4D42G, G4D42J, G8H8C, G8H8L, G8H8M, G8H8N, G8H8O,G8H8d, G8H8e, G8H8P, G9E3C, G9E3R, G9E3S, G9E3T, G9E3a, and G9E3U) were constructed by transforming the relative plasmid to their parental strain, separately.	

Supplementary Table 6. Sequences of DNA modules used in this work

DNA fragment	Sequence
$P_{CYC1core(-)}$	GAGCTC<br>ACGGCGGCCAAGCACGCGGGGATAATGAACTAGATTTCCGTGTGAGACGA<br>CATCGTCGATACTGATGTAATCAAGTTC<br><br>CTACCTGAATCTAAGATTCCCGGGCCGGCAATCCTCGAGCA<br><br>GATCCGCCAGGCGTGTATATATAGCGTGGATGGCCAGGCAACTTTAGTGCT<br>GACACATACAGGCATATATATATGTGTGCGACGACACATGATCATATGGC<br>ATGCATGTGCTCTGTATGTATATAAAACTCTTGTTTTCTTCTTTTCTCTAAA<br>TATTCTTTCCTTATACATTAGGACCTTTGCAGCATAAATTACTATACTTCTA<br>TAGACACACAAAC<br>GGATCCAAA
$P_{CYC1core+4\times[Z268]}$	GAGCTC<br><br>ACGGCGGCCAAGCACGCGGGGATAATGAACTAGATTTCCGTGTGAGACGA<br>CATCGTCGATACTGATGTAATCAAGTTC<br><br>CTACCTGAATCTAAGATTGGGCCC**GCGTGGGCG**AATTGGT**GCGTGGGCG**<br>TAAGCGGGGGCGG**GCGTGGGCG**TA**GCGTGGGCG**GCCGGCAATCCTCGA<br>GCA<br><br>GATCCGCCAGGCGTGTATATATAGCGTGGATGGCCAGGCAACTTTAGTGCT<br>GACACATACAGGCATATATATATGTGTGCGACGACACATGATCATATGGC<br>ATGCATGTGCTCTGTATGTATATAAAACTCTTGTTTTCTTCTTTTCTCTAAA<br>TATTCTTTCCTTATACATTAGGACCTTTGCAGCATAAATTACTATACTTCTA<br>TAGACACACAAAC<br><br>GGATCCAAA
$P_{TEF1(-)}$	GAGCTC<br>CAGAAAGCGACCACCCAACTTTGGCTGATAATAGCGTATAAACAATGCAT<br>ACTTTGTACGTTCAAAATACAATGCAGTAGATATATTTATGCATATTACAT<br>ATAATACATATCACATAGGAAGCAACAGGCGCGTTGGACTTTTAATTTTCG<br>AGGACCGCGAATCCTTACATCACACCCAATCCCCCACAAGTGATCCCCCAC<br>ACACCATAGCTTCAAAATGTTTCTACTCCTTTTTTACTCTTCCAGATTTTCT<br>CGGACTCCGCGCATCGCCGTACCACTTCAAAACACCCAAGCACAGCATAC<br>TAAATTTCCCCTCTTTCTTCCTCTAGGGTGTCGTTAATTACCCGTACTAAAG<br>GTTTGGAAAAGAAAAAAGAGACCGCCTCGTTTCTTTTTCTTCGTCGAAAAA<br>GGCAATAAAAATTTTTATCACGTT<br><br>CTACCTGAATCTAAGATTCCCGGGCCGGCAATCCTCGAGCA<br><br>GATTCTTTTTCTTGAAAATTTTTTTTTTTGATTTTTTTCTCTTTCGATGACCT<br>CCCATTGATATTTAAGTTAATAAACGGTCTTCAATTTCTCAAGTTTCAGTTT<br>CATTTTTCTTGTTCTATTACAACTTTTTTTACTTCTTGCTCATTAGAAAGAA<br>AGCATAGCAATCTAATCTAAGTTTTAATTACAAA<br>GGATCCAAA
$P_{TEF1+4\times[Z268]}$	GAGCTC<br>CAGAAAGCGACCACCCAACTTTGGCTGATAATAGCGTATAAACAATGCAT<br>ACTTTGTACGTTCAAAATACAATGCAGTAGATATATTTATGCATATTACAT<br>ATAATACATATCACATAGGAAGCAACAGGCGCGTTGGACTTTTAATTTTCG<br>AGGACCGCGAATCCTTACATCACACCCAATCCCCCACAAGTGATCCCCCAC

	ACACCATAGCTTCAAAATGTTTCTACTCCTTTTTTACTCTTCCAGATTTTCT<br>CGGACTCCGCGCATCGCCGTACCACTTCAAAACACCCAAGCACAGCATAC<br>TAAATTTCCCCTCTTTCTTCCTCTAGGGTGTCGTTAATTACCCGTACTAAAG<br>GTTTGGAAAAGAAAAAAGAGACCGCCTCGTTTCTTTTTCTTCGTCGAAAAA<br>GGCAATAAAAATTTTTATCACGTT<br><br>CTACCTGAATCTAAGATTGGGCCC**GCGTGGGCG**AATTGGT**GCGTGGGCG**<br>TAAGCGGGGGCGG**GCGTGGGCG**TA**GCGTGGGCG**GCCGGCAATCCTCGA<br>GCA<br><br>GATTCTTTTTCTTGAAAATTTTTTTTTTTGATTTTTTTCTCTTTCGATGACCT<br>CCCATTGATATTTAAGTTAATAAACGGTCTTCAATTTCTCAAGTTTCAGTTT<br>CATTTTTCTTGTTCTATTACAACTTTTTTTACTTCTTGCTCATTAGAAAGAA<br>AGCATAGCAATCTAATCTAAGTTTTAATTACAAA<br><br>GGATCCAAA
$P_{TEF1+4\times[PZ4]}$	GAGCTC<br>CAGAAAGCGACCACCCAACTTTGGCTGATAATAGCGTATAAACAATGCAT<br>ACTTTGTACGTTCAAAATACAATGCAGTAGATATATTTATGCATATTACAT<br>ATAATACATATCACATAGGAAGCAACAGGCGCGTTGGACTTTTAATTTTCG<br>AGGACCGCGAATCCTTACATCACACCCAATCCCCCACAAGTGATCCCCCAC<br>ACACCATAGCTTCAAAATGTTTCTACTCCTTTTTTACTCTTCCAGATTTTCT<br>CGGACTCCGCGCATCGCCGTACCACTTCAAAACACCCAAGCACAGCATAC<br>TAAATTTCCCCTCTTTCTTCCTCTAGGGTGTCGTTAATTACCCGTACTAAAG<br>GTTTGGAAAAGAAAAAAGAGACCGCCTCGTTTCTTTTTCTTCGTCGAAAAA<br>GGCAATAAAAATTTTTATCACGTT<br><br>CTACCTGAATCTAAGATTGGGCCCTA<br>**GAGTGAGACGTT** aattggt<br>A**GAGTGAGACGTT** taagcgggggcggC TA**GAGTGAGACGTT** gggctta<br>**GAGTGAGACGTT**<br>GCCGGCAATCCTCGAGCA<br><br>GATTCTTTTTCTTGAAAATTTTTTTTTTTGATTTTTTTCTCTTTCGATGACCT<br>CCCATTGATATTTAAGTTAATAAACGGTCTTCAATTTCTCAAGTTTCAGTTT<br>CATTTTTCTTGTTCTATTACAACTTTTTTTACTTCTTGCTCATTAGAAAGAA<br>AGCATAGCAATCTAATCTAAGTTTTAATTACAAA<br><br>GGATCCAAA
$P_{TEF1+4\times[TetO]}$	GAGCTC<br>CAGAAAGCGACCACCCAACTTTGGCTGATAATAGCGTATAAACAATGCAT<br>ACTTTGTACGTTCAAAATACAATGCAGTAGATATATTTATGCATATTACAT<br>ATAATACATATCACATAGGAAGCAACAGGCGCGTTGGACTTTTAATTTTCG<br>AGGACCGCGAATCCTTACATCACACCCAATCCCCCACAAGTGATCCCCCAC<br>ACACCATAGCTTCAAAATGTTTCTACTCCTTTTTTACTCTTCCAGATTTTCT<br>CGGACTCCGCGCATCGCCGTACCACTTCAAAACACCCAAGCACAGCATAC<br>TAAATTTCCCCTCTTTCTTCCTCTAGGGTGTCGTTAATTACCCGTACTAAAG<br>GTTTGGAAAAGAAAAAAGAGACCGCCTCGTTTCTTTTTCTTCGTCGAAAAA<br>GGCAATAAAAATTTTTATCACGTT<br><br>CTACCTGAATCTAAGATTGGGCCCTA<br>ACTCTATCATTGATAGAGT aattggt A TCCCTATCAGTGATAGAGA taagcgggggcgg TA<br>TCCCTATCAGTGATAGAGA gggctta ACTCTATCATTGATAGAGT

	GCCGGCAATCCTCGAGCA<br><br>GATTCTTTTTCTTGAAAATTTTTTTTTTGATTTTTTTCTCTTTCGATGACCT<br>CCCATTGATATTTAAGTTAATAAACGGTCTTCAATTTCTCAAGTTTCAGTTT<br>CATTTTTCTTGTTCTATTACAACTTTTTTACTTCTTGCTCATTAGAAAGAA<br>AGCATAGCAATCTAATCTAAGTTTTAATTACAAA<br><br>GGATCCAAA
Zif268-SV40^{NLS}-VP16^{A}	ATG<br>GGTACTAGACCATACGCTTGTCCAGTTGAATCTTGTGATAGAAGATTCTCC<br>AGATCCGATGAATTGACCAGACATATTAGAATCCATACCGGTCAAAAGCC<br>ATTCCAATGTAGAATCTGTATGAGGAACTTCTCCAGGTCTGATCATTTGAC<br>TACTCATATCAGAACCCACACTGGTGAAAAACCATTCGCTTGTGATATTTG<br>CGGTAGAAAGTTTGCTAGATCCGACGAAAGAAAAAGACATACCAAAATTC<br>AT<br>TCTAGA<br>GGTGGTGGTACTCCAGCTGCTGCTTCTACTTTGGAA<br>GTCGAC<br>TGTACCCCACCTAAGAAGAAAAGAAAGGTT<br>GGTACC<br>GAATTGCATTTGGATGGTGAAGATGTTGCTATGGCTCATGCTGATGCTTTG<br>GATGATTTTGACTTGGATATGTTAGGTGACGGTGACTCTCCAGGTCCAGGT<br>TTTACTCCTCATGATTCTGCTCCTTATGGTGCTTTGGATATGGCTGATTTTG<br>AATTTGAACAAATGTTTACTGATGCCTTGGGTATTGATGAATACGGTGGTT<br>AA
*Zif268-SV40^{NLS}-10*WD*	ATG<br>GGTACTAGACCATACGCTTGTCCAGTTGAATCTTGTGATAGAAGATTCTCC<br>AGATCCGATGAATTGACCAGACATATTAGAATCCATACCGGTCAAAAGCC<br>ATTCCAATGTAGAATCTGTATGAGGAACTTCTCCAGGTCTGATCATTTGAC<br>TACTCATATCAGAACCCACACTGGTGAAAAACCATTCGCTTGTGATATTTG<br>CGGTAGAAAGTTTGCTAGATCCGACGAAAGAAAAAGACATACCAAAATTC<br>AT<br>TCTAGA<br>GGTGGTGGTACTCCAGCTGCTGCTTCTACTTTGGAA<br>GTCGAC<br>TGTACCCCACCTAAGAAGAAAAGAAAGGTT<br>GGTACC<br>TGGGATTGGGATTGGGACTGGGATTGGGACTGGGACTGGGATTGGGATTG<br>GGACTGGGAC<br>TAA
Zif268-SV40^{NLS}-GCN4^{A}	ATG<br>GGTACTAGACCATACGCTTGTCCAGTTGAATCTTGTGATAGAAGATTCTCC<br>AGATCCGATGAATTGACCAGACATATTAGAATCCATACCGGTCAAAAGCC<br>ATTCCAATGTAGAATCTGTATGAGGAACTTCTCCAGGTCTGATCATTTGAC<br>TACTCATATCAGAACCCACACTGGTGAAAAACCATTCGCTTGTGATATTTG<br>CGGTAGAAAGTTTGCTAGATCCGACGAAAGAAAAAGACATACCAAAATTC<br>AT<br>TCTAGA<br>GGTGGTGGTACTCCAGCTGCTGCTTCTACTTTGGAA<br>GTCGAC<br>TGTACCCCACCTAAGAAGAAAAGAAAGGTT<br>GGTACC

	ACTCCAATGTTTGAGTATGAAAACCTAGAAGACAACTCTAAAGAATGGAC<br>ATCCTTGTTTGACAATGACATTCCAGTTACCACTGACGATGTTTCATTGGCT<br>GATAAGGCAATTGAATCC<br>TAA
Zif268-SV40^{NLS}-MED3	ATG<br>GGTACTAGACCATACGCTTGTCCAGTTGAATCTTGTGATAGAAGATTCTCC<br>AGATCCGATGAATTGACCAGACATATTAGAATCCATACCGGTCAAAAGCC<br>ATTCCAATGTAGAATCTGTATGAGGAACTTCTCCAGGTCTGATCATTTGAC<br>TACTCATATCAGAACCCACACTGGTGAAAAACCATTCGCTTGTGATATTTG<br>CGGTAGAAAGTTTGCTAGATCCGACGAAAGAAAAAGACATACCAAAATTC<br>AT<br>TCTAGA<br>GGTGGTGGTACTCCAGCTGCTGCTTCTACTTTGGAA<br>GTCGAC<br>TGTACCCCACCTAAGAAGAAAAGAAAGGTT<br>GGTACC<br>GACTCGATTATACCGGCAGGCGTCAAGCTAGATGATTTACAAGTGATATTG<br>GCTAAGAACGAGAATGAAACCAGGGACAAAGTGTGTAAGCAGATCAACG<br>AAGCACGCGATGAAATTCTGCCGTTGCGATTGCAGTTCAATGAATTCATAC<br>AGATAATGGCAAACATAGACCAAGAAGGATCCAAGCAGGCTGACCGTATG<br>GCCAAATACCTACATATCAGAGACAAGATTTTGCAACTGAACGATAGATT<br>CCAGACTTTGTCTTCGCATTTAGAAGCACTGCAACCTTTATTCAGCACTGT<br>ACCAGAATACTTGAAGACTGCAGATAACAGGGACAGAAGTTTCCAGCTTT<br>TGGAACCGTTGAGTACTTATAACAAAAATGGCAACGCCGTTTGCTCGACA<br>GCAACTGTTGTAAGCACCAACCACTCTGCGGCCGCTTCGACACCCACCACA<br>ACTGCCACCCCTCATGCAAATCCCATCACACATGCGCACTCGCTTTCGAAT<br>CCAAATAGCACCGCTACAATGCAACATAATCCCCTGGCCGGCAAGAGAGG<br>TCCAAAAAGCGGTAGTACTATGGGAACTCCAACCGTACATAATAGCACTG<br>CAGCTGCACCGATTGCGGCACCTAAAAAGCCAAGAAAACCAAGACAAACC<br>AAGAAGGCAAAGGCTCAAGCTCAGGCTCAAGCGCAGGCTCAAGCGCAGGT<br>CTACGCACAGCAGTCGACTGTCCAGACGCCAATAACAGCATCGATGGCAG<br>CCGCGCTACCCAACCCAACTCCTAGCATGATTAACAGCGTTTCGCCCACAA<br>ATGTTATGGGCACGCCGCTGACCAACATGATGTCTCCCATGGGGAACGCAT<br>ACTCAATGGGAGCTCAAAACCAAGGGGGACAAGTATCTATGTCACAATTC<br>AATGGCAGCGGCAATGGATCCAATCCAAATACAAATACAAACTCCAATAA<br>CACCCCGCTACAGTCACAATTAAACCTAAACAATCTAACCCCTGCTAATAT<br>CCTAAATATGAGCATGAATAACGATTTCCAGCAGCAGCAGCAACAGCAAC<br>AGCAGCAGCAGCAGCCTCAACCACAGTACAATATGAATATGGGCATGAAT<br>AACATGAATAACGGGGGGAAAGAACTGGATTCTCTAGACCTGAACAATCT<br>GGAATTAGGTGGTCTGAACATGGATTTCTTG<br>TAA
Zif268-SV40^{NLS}-MED15	ATG<br>GGTACTAGACCATACGCTTGTCCAGTTGAATCTTGTGATAGAAGATTCTCC<br>AGATCCGATGAATTGACCAGACATATTAGAATCCATACCGGTCAAAAGCC<br>ATTCCAATGTAGAATCTGTATGAGGAACTTCTCCAGGTCTGATCATTTGAC<br>TACTCATATCAGAACCCACACTGGTGAAAAACCATTCGCTTGTGATATTTG<br>CGGTAGAAAGTTTGCTAGATCCGACGAAAGAAAAAGACATACCAAAATTC<br>AT<br>TCTAGA<br>GGTGGTGGTACTCCAGCTGCTGCTTCTACTTTGGAA<br>GTCGAC<br>TGTACCCCACCTAAGAAGAAAAGAAAGGTT<br>GGTACC<br>TCTGCTGCTCCTGTCCAAGACAAAGACACTCTGTCCAATGCCGAGCGTGCG<br>AAGAACGTCAACGGGTTGCTTCAGGTGCTCATGGACATTAACACTCTGAAC

	GGAGGGAGCTCCGACACTGCTGATAAGATAAGAATTCATGCCAAAAACTT<br>CGAGGCAGCTTTGTTCGCAAAGAGCTCTTCAAAGAAAGAATACATGGACA<br>GCATGAACGAAAAAGTTGCTGTCATGCGCAACACGTACAATACGAGGAAA<br>AACGCCGTTACTGCTGCTGCCGCTAATAACAACATTAAACCCGTGGAACA<br>GCACCATATTAACAACTTGAAAAATTCTGGCAACAGCGCCAATAATATGA<br>ATGTCAATATGAATCTAAACCCACAGATGTTCTTGAATCAGCAGGCTCAGG<br>CAAGGCAACAGGTTGCGCAACAATTAAGAAATCAACAACAACAACAACA<br>ACAGCAGCAGCAGCAACAGAGGCGTCAATTGACTCCTCAACAACAACAAT<br>TAGTGAACCAGATGAAAGTGGCACCTATTCCCAAACAATTACTGCAAAGA<br>ATTCCTAACATTCCACCCAATATCAACACCTGGCAGCAGGTCACTGCTTTG<br>GCTCAACAAAAGCTATTGACACCTCAGGATATGGAAGCTGCGAAGGAAGT<br>CTACAAGATTCACCAGCAGTTGCTATTCAAAGCAAGGCTACAGCAACAAC<br>AAGCACAGGCTCAAGCACAAGCTAATAACAACAACAACGGCCTCCCCCAA<br>AATGGTAATATTAACAATAACATAAATATTCCTCAACAGCAGCAAATGCA<br>ACCTCCCAATTCAAGTGCGAACAACAACCCTTTGCAACAGCAATCATCACA<br>AAATACCGTACCAAACGTCCTCAACCAAATTAACCAAATCTTTTCTCCAGA<br>GGAGCAACGCAGCTTATTACAAGAAGCCATCGAAACCTGCAAGAATTTTG<br>AAAAAACACAATTGGGTAGTACGATGACGGAACCTGTCAAGCAAAGTTTT<br>ATTAGGAAATACATTAACCAAAAGGCCCTGAGAAAAATCCAAGCTTTGAG<br>AGATGTTAAGAACAACAATAACGCTAACAACAACGGCTCGAACCTTCAGA<br>GAGCCCAAAATGTCCCTATGAATATCATCCAACAACAACAACAACAGAAC<br>ACGAACAATAATGACACCATTGCCACTTCTGCTACACCTAATGCTGCCGCT<br>TTCTCTCAGCAACAGAACGCAAGTTCTAAATTATATCAGATGCAACAACAG<br>CAACAAGCTCAAGCTCAAGCTCAAGCTCAAGCTCAGGCACAGGCTCAAGC<br>ACAAGCTCAAGCACAGGCGGCACAAGCGGCGCAAGCGCAAGCACAAGCA<br>CAAGCACAAGCACAAGCACAGGCACAGGCACAGGCACAAGCCCAGGCGC<br>AGGCCCAAGCCCAAGCCCAAGCACAAGCACAAGCACACGCTCAGCACCAG<br>CCCTCCCAACAACCACAACAAGCTCAGCAACAACCTAACCCACTACATGG<br>GTTGACACCTACTGCAAAGGATGTCGAAGTAATTAAGCAATTGTCCTTGGA<br>TGCTTCTAAGACCAACCTAAGGCTTACGGACGTAACAAATTCTTTATCCAA<br>TGAAGAAAAGGAAAAAATTAAAATGAAGTTAAAGCAAGGTCAAAAGCTTT<br>TTGTTCAGGTGAGTAATTTCGCCCCACAAGTCTACATCATCACAAAGAATG<br>AAAACTTCTTGAAGGAAGTTTTTCAGTTAAGAATCTTTGTAAAAGAGATCC<br>TAGAAAAATGTGCCGAGGGTATATTTGTTGTTAAATTAGACACCGTTGACA<br>GGTTAATTATTAAGTATCAAAAATATTGGGAAAGTATGAGAATTCAAATTT<br>TAAGAAGACAAGCTATTTTAAGACAACAACAGCAAATGGCAAACAACAAT<br>GGGAACCCAGGCACTACTTCTACTGGAAACAATAATAATATTGCAACTCA<br>GCAAAATATGCAACAGTCACTACAGCAAATGCAGCATTTACAGCAATTGA<br>AAATGCAGCAGCAACAACAACAGCAGCAACAACAACAGCAGCAACAACA<br>ACAGCAGCAACAACAACAGCAACAGCACATATATCCCTCCTCGACTCCTG<br>GTGTGGCTAATTATTCGGCAATGGCTAATGCACCCGGTAACAATATCCCAT<br>ATATGAACCATAAAAATACCTCTAGCATGGATTTTTTGAACTCTATGGAAA<br>ATACACCAAAAGTTCCCGTATCCGCTGCGGCAACCCCATCACTGAACAAG<br>ACGATCAACGGTAAGGTGAATGGCAGGACAAAATCTAATACGATACCTGT<br>TACCAGCATTCCATCAACAAATAAGAAACTATCAATTTCAAATGCCGCTAG<br>TCAACAACCCACTCCTAGGTCTGCATCAAATACCGCTAAGTCAACCCCAAA<br>TACAAATCCTTCTCCACTGAAAACCCAAACTAAAAATGGCACACCAAACC<br>CCAATAATATGAAGACTGTACAGTCTCCTATGGGTGCACAACCATCATATA<br>ATAGTGCCATTATAGAGAATGCATTCAGAAAGGAAGAACTCTTGTTAAAA<br>GATTTGGAAATCAGGAAGTTGGAGATATCTTCTCGTTTTAAACATCGCCAA<br>GAAATTTTCAAAGATTCTCCTATGGATTTGTTTATGAGTACGCTGGGTGAT<br>TGCTTAGGTATCAAAGATGAAGAGATGCTTACGTCATGCACTATCCCAAAG<br>GCTGTGGTTGATCACATCAACGGCTCTGGCAAAAGGAAGCCTACAAAAGC<br>GGCTCAAAGGGCTCGCGATCAAGATTCCATTGACATTTCCATAAAAGACA<br>ACAAATTGGTTATGAAGAGTAAATTCAATAAGAGCAATAGGTCGTATTCG

	ATAGCGTTGTCCAATGTAGCTGCTATATTCAAGGGTATCGGTGGTAACTTT<br>AAAGACTTGTCCACTTTGGTTCATTCATCATCGCCGTCCACATCTTCTAATA<br>TGGATGTCGGCAACCCAAGAAAAAGAAAAGCCAGCGTATTAGAAATAAGC<br>CCGCAAGATTCGATAGCCTCGGTGCTATCACCAGATTCAAATATAATGAGT<br>GATTCTAAAAAAATTAAAGTAGATTCTCCTGATGACCCATTCATGACAAAA<br>TCAGGAGCCACAACTAGTGAAAAACAAGAAGTAACAAATGAAGCTCCATT<br>TTTAACTTCTGGGACTAGTTCAGAACAATTCAATGTATGGGATTGGAATAA<br>TTGGACAAGTGCTACT<br>TAA
P$_{Sk.GAL2}$<br>(2*PZ4)	TAAACCAATTTTATTTGAACTTGCCCCGCTAATCGTACAGACGTACCAGAT<br>CCAGGTCATAACGGTGCCATTAGAAGGCCGTAGAATAGACCCACTAAAGC<br>TATCACAGGCAACCTTGCATCCAAGTCTAATTAAACGGAGCAGAATCTGTT<br>CCAGTAATGTATAAAGAATTAACTACCGGAAAAGCGTCTTCCGGGGCGGC<br>TCAACCGGATTACATTCTTTTTTCTTTTTTGCGGCCAGCATTCGAGTCACGT<br>GCTCAAATACTGAGGAAGGGGTTCTTCCGGTAGGCACCGGCGCTCACTCTT<br>CCGTGCGGCACACTCCCCGCAGTTCAGGGGCCATGTGCCTGTGAGAGTATT<br>TCCGCAGGCCATAGTTCCGGGGTGGACCACTCCGAGCTAATGCTAAAGTA<br>AGCCCTTCCCAACTTGAAATAGGAACAAAAGTCAATTATGCTCCTCCTGTA<br>AAGCTAAAC**TAGAGTGAGACGTT**TTTA**GAGTGAGACGTT**GTTAGCAGTC<br>GTTGTTCAAGGGAGGGATGAGTTGAGGAATAAAATGCGGAAAAAGTGTGG<br>GTTAGACAGACATATATAAACTCTAGCCATTCGGCTGAAATATATCTGAGT<br>TCATTTGTGATAACTTTTTTGATTACTTGTTCATATATCCACAAGAACAACG<br>ACTATTAAACAATAGAAAATAGTATAATAAAAAAAGTTTTTTACA
P$_{Sk.GAL2}$<br>(3*PZ4)	TAAACCAATTTTATTTGAACTTGCCCCGCTAATCGTACAGACGTACCAGAT<br>CCAGGTCATAACGGTGCCATTAGAAGGCCGTAGAATAGACCCACTAAAGC<br>TATCACAGGCAACCTTGCATCCAAGTCTAATTAAACGGAGCAGAATCTGTT<br>CCAGTAATGTATAAAGAATTAACTACCGGAAAAGCGTCTTCCGGGGCGGC<br>TCAACCGGATTACATTCTTTTTTCTTTTTTGCGGCCAGCATTCGAGTCACGT<br>GCTCAAATACTGAGGAAGGGGTTCTTCCGGTAGGCACCGGCGCTCACTCTT<br>CCGTGCGGCACACTCCCCGCAGTTCAGGGGCCATGTGCCTGTGAGAGTATT<br>TCCGCAGGCCATAGTTCCGGGGTGGACCACTCCGAGCTAATGCTAAAGTA<br>AGCCCTTCCCAACTTGAAATAGGAACAAAAGTCAATTATGCTCCTCCTGTA<br>AAGCTAAAC**TAGAGTGAGACGTT**TTTA**GAGTGAGACGTT**GTTA**GAGTG**<br>**AGACGTT**GTTCAAGGGAGGGATGAGTTGAGGAATAAAATGCGGAAAAAG<br>TGTGGGTTAGACAGACATATATAAACTCTAGCCATTCGGCTGAAATATATC<br>TGAGTTCATTTGTGATAACTTTTTTGATTACTTGTTCATATATCCACAAGAA<br>CAACGACTATTAAACAATAGAAAATAGTATAATAAAAAAAGTTTTTTACA
P$_{Sk.GAL2}$<br>(2*PZ4’)	TAAACCAATTTTATTTGAACTTGCCCCGCTAATCGTACAGACGTACCAGAT<br>CCAGGTCATAACGGTGCCATTAGAAGGCCGTAGAATAGACCCACTAAAGC<br>TATCACAGGCAACCTTGCATCCAAGTCTAATTAAACGGAGCAGAATCTGTT<br>CCAGTAATGTATAAAGAATTAACTACCGGAAAAGCGTCTTCCGGGGCGGC<br>TCAACCGGATTACATTCTTTTTTCTTTTTTGCGGCCAGCATTCGAGTCACGT<br>GCTCAAATACTGAGGAAGGGGTTCTTCCGGTAGGCACCGGCGCTCACTCTT<br>CCGTGCGGCACACTCCCCGCAGTTCAGGGGCCATGTGCCTGTGAGAGTATT<br>TCCGCAGGCCATAGTTCCGGGGTGGACCACTCCGAGCTAATGCTAAAGTA<br>AGCCCTTCCCAACTTGAAATAGGAACAAAAGTCAATTATCTACCTGAATCT<br>AAGATTGGGCCC**TAGAGTGAGACGTT**GCCGGCAATCCTCGAGCAGCTCCT<br>CCTGTAAAGCTAAACGTGTAAAGATTT**TAGAGTGAGACGTT**GTTAGCAGT<br>CGTTGTTCAAGGGAGGGATGAGTTGAGGAATAAAATGCGGAAAAAGTGTG<br>GGTTAGACAGACATATATAAACTCTAGCCATTCGGCTGAAATATATCTGAG<br>TTCATTTGTGATAACTTTTTTGATTACTTGTTCATATATCCACAAGAACAAC<br>GACTATTAAACAATAGAAAATAGTATAATAAAAAAAGTTTTTTACA

$P_{Sk.GAL2}$<br>(4*PZ4)	TAAACCAATTTTATTTGAACTTGCCCCGCTAATCGTACAGACGTACCAGAT<br>CCAGGTCATAACGGTGCCATTAGAAGGCCGTAGAATAGACCCACTAAAGC<br>TATCACAGGCAACCTTGCATCCAAGTCTAATTAAACGGAGCAGAATCTGTT<br>CCAGTAATGTATAAAGAATTAACTACCGGAAAAGCGTCTTCCGGGGCGGC<br>TCAACCGGATTACATTCTTTTTTCTTTTTTGCGGCCAGCATTCGAGTCACGT<br>GCTCAAATACTGAGGAAGGGGTTCTTCCGGTAGGCACCGGCGCTCACTCTT<br>CCGTGCGGCACACTCCCCGCAGTTCAGGGGCCATGTGCCTGTGAGAGTATT<br>TCCGCAGGCCATAGTTCCGGGGTGGACCACTCCGAGCTAATGCTAAAGTA<br>AGCCCTTCCCAACTT<br>GAAATAGGAACAAAAGTCAATTAT<br>CTACCTGAATCTAAGATTGG<br>GCCCTA<br>**GAGTGAGACGTT** aattggt A**GAGTGAGACGTT** taagcgggggcgg<br>TA**GAGTGAGACGTT** gggctta GAGTGAACGTT<br>GC<br>CGGCAATCCTCGAGCA<br>GCTCCTCCTGTAAAGCTAAAC<br>GTGTAAAGATTTTAGAGTGAGACGTTGTTAGCAGTCGTTGTTCAAGGGAGG<br>GATGAGTTGAGGAATAAAATGCGGAAAAAGTGTGGGTTAGACAGACATAT<br>ATAAACTCTAGCCATTCGGCTGAAATATATCTGAGTTCATTTGTGATAACT<br>TTTTTGATTACTTGTTCATATATCCACAAGAACAACGACTATTAAACAATA<br>GAAAATAGTATAATAAAAAAAGTTTTTACA
$P_{Sk.GAL2}$<br>(5*PZ4)	TAAACCAATTTTATTTGAACTTGCCCCGCTAATCGTACAGACGTACCAGAT<br>CCAGGTCATAACGGTGCCATTAGAAGGCCGTAGAATAGACCCACTAAAGC<br>TATCACAGGCAACCTTGCATCCAAGTCTAATTAAACGGAGCAGAATCTGTT<br>CCAGTAATGTATAAAGAATTAACTACCGGAAAAGCGTCTTCCGGGGCGGC<br>TCAACCGGATTACATTCTTTTTTCTTTTTTGCGGCCAGCATTCGAGTCACGT<br>GCTCAAATACTGAGGAAGGGGTTCTTCCGGTAGGCACCGGCGCTCACTCTT<br>CCGTGCGGCACACTCCCCGCAGTTCAGGGGCCATGTGCCTGTGAGAGTATT<br>TCCGCAGGCCATAGTTCCGGGGTGGACCACTCCGAGCTAATGCTAAAGTA<br>AGCCCTTCCCAACTT<br>GAAATAGGAACAAAAGTCAATTAT<br>CTACCTGAATCTAAGATTGG<br>GCCCTA<br>**GAGTGAGACGTT** aattggt A**GAGTGAGACGTT** taagcgggggcgg<br>TA**GAGTGAGACGTT** gggctta **GAGTGAGACGTT**<br>GC<br>CGGCAATCCTCGAGCA<br>GCTCCTCCTGTAAAGCTAAAC<br>GTGTAAAGATTTTAGAGTGAGACGTTGTTAGCAGTCGTTGTTCAAGGGAGG<br>GATGAGTTGAGGAATAAAATGCGGAAAAAGTGTGGGTTAGACAGACATAT<br>ATAAACTCTAGCCATTCGGCTGAAATATATCTGAGTTCATTTGTGATAACT<br>TTTTTGATTACTTGTTCATATATCCACAAGAACAACGACTATTAAACAATA<br>GAAAATAGTATAATAAAAAAAGTTTTTACA
$P_{Sk.GAL2}$<br>(4*Z268)	TAAACCAATTTTATTTGAACTTGCCCCGCTAATCGTACAGACGTACCAGAT<br>CCAGGTCATAACGGTGCCATTAGAAGGCCGTAGAATAGACCCACTAAAGC<br>TATCACAGGCAACCTTGCATCCAAGTCTAATTAAACGGAGCAGAATCTGTT<br>CCAGTAATGTATAAAGAATTAACTACCGGAAAAGCGTCTTCCGGGGCGGC<br>TCAACCGGATTACATTCTTTTTTCTTTTTTGCGGCCAGCATTCGAGTCACGT<br>GCTCAAATACTGAGGAAGGGGTTCTTCCGGTAGGCACCGGCGCTCACTCTT<br>CCGTGCGGCACACTCCCCGCAGTTCAGGGGCCATGTGCCTGTGAGAGTATT

	TCCGCAGGCCATAGTTCCGGGGTGGACCACTCCGAGCTAATGCTAAAGTA AGCCCTTCCCAACTTGAAATAGGAACAAAAGTCAATTAT<br>CTACCTGAATCTAAGATTGGGCCC<br>**GCGTGGGCG**aattggt**GCGTGGGCG**taagcgggggcgg**GCGTGGGCG**ta**GCGTG GGCG**<br>GCCGGCAATCCTCGAGCA<br>GCTCCTCCTGTAAAGCTAAACGTGTAAAGATTTTAGAGTGAGACGTTGTTA GCAGTCGTTGTTCAAGGGAGGGATGAGTTGAGGAATAAAATGCGGAAAAA GTGTGGGTTAGACAGACATATATAAACTCTAGCCATTCGGCTGAAATATAT CTGAGTTCATTTGTGATAACTTTTTTGATTACTTGTTCATATATCCACAAGA ACAACGACTATTAAACAATAGAAAATAGTATAATAAAAAAAGTTTTTAC A
ZifPZ43	GGTACTAGACCATACGCTTTTAAGTGTCCAGTTTGTGGTAAAGCCTTCAGA CATTCTTCATCCTTGGTTAGACATCAAAGAACTCATACTGGTCAAAAGCCA TACAAGTGTAAGCAATGTGGAAAGGCTTTTGGTTGTCCATCTAATTTGAGA AGGCATGGTAGAACCCATACAGGTGAAAAACCTTATGAATGTCACGATTG TGGCAAGTCTTTCAGACAATCTACTCATTTGACTCAGCATAGAAGAATCCA C
TetR	TCCAGATTGGATAAGTCCAAGGTTATTAACTCTGCCTTGGAATTGTTGAAC GAAGTTGGTATTGAAGGTTTGACCACTAGAAAGTTGGCTCAAAAATTGGG TGTTGAACAACCTACCTTGTACTGGCATGTTAAGAACAAAAGAGCTTTGTT GGACGCTTTGGCCATTGAAATGTTGGATAGACATCATACCCATTTCTGTCC ATTGGAAGGTGAATCATGGCAAGATTTCTTGAGAAACAACGCCAAGTCTTT CAGATGTGCTTTGTTGTCTCATAGAGATGGTGCTAAAGTTCACTTGGGTAC TAGACCAACTGAAAAGCAATACGAAACTTTGGAAAACCAGTTGGCTTTCTT GTGTCAACAAGGTTTCTCTTTAGAAAACGCCTTGTATGCTTTGTCTGCTGTT GGTCATTTTACCTTGGGTTGTGTTTTGGAAGATCAAGAACATCAAGTCGCC AAAGAAGAAAGGGAAACTCCAACTACTGATTCTATGCCACCATTATTGAG ACAAGCCATTGAGTTGTTTGATCATCAAGGTGCTGAACCAGCTTTTTTGTTT GGTTTGGAATTGATCATCTGCGGTTTGGAGAAACAGTTGAAATGTGAATCT GGCTCC
H.Degron	ATG<br>CAGATTTTCGTCAAGACTTTGACCGGTAAAACCATAACATTGGAAGTTGAA TCTTCCGATACCATCGACAACGTTAAGTCGAAAATTCAAGACAAGGAAGG TATCCCTCCAGATCAACAAAGATTGATCTTTGCCGGTAAGCAGCTAGAAGA CGGTAGAACGCTGTCTGATTACAACATTCAGAAGGAGTCCACCTTACATCT TGTGCTAAGGCTAAGAGGTGGT<br><br>AGACATGGTTCTGGTATCATGGTT<br><br>AGACCATTGAACTGTATCGTTGCTGTTTCTCAAAACATGGGTATTGGAAAG AATGGTGACTTGCCTTGGCCACCATTGAGAAATGAGTTTAAGTACTTCCAG AGAATGACCACCACTTCTTCAGTTGAAGGTAAACAAAACTTGGTCATCATG GGTAGAAAGACCTGGTTTTCTATTCCAGAAAAGAACAGGTTGTTGAAGGA CAGAATCAACATCGTTTTGTCCAGGGAATTGAAAGAACCACCAAGAGGTG CTCATTTCTTGGCAAAATCTTTGGATGATGCCTTGAGATTGATCGAACAAC CAGAATTGGCTTCCAAGGTTGATATGGTTTGGATTGTCGGTGGTTCATCTG TTTATCAAGAAGCCATGAATCAACCAGGTCATTTGAGGTTGTTCGTTACCA GAATCATGCAAGAGTTTGAGTCTGATACTTTCTTCCCTGAAATTGACTTGG GCAAGTATAAGTTGTTGCCAGAATATCCAGGTGTCTTGTCTGAAGTTCAAG AAGAAAAGGGTATCAAGTACAAGTTCGAAGTCTACGAAAAGAAGGAT

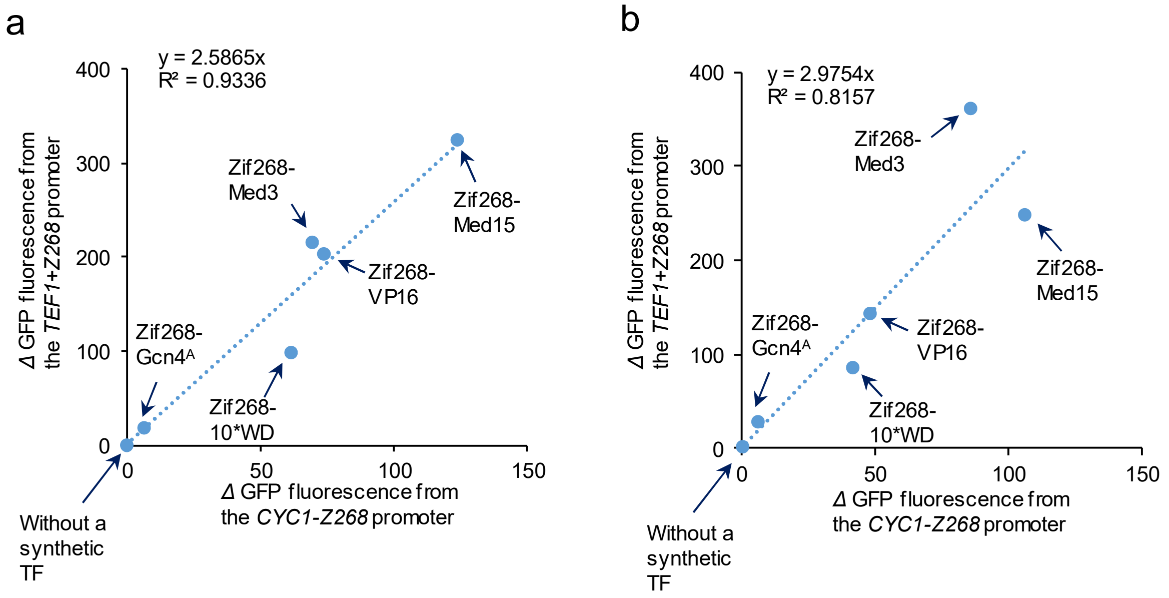

Supplementary Figure 1. The correlation of *trans*-activation effects from Zif268-derivative artificial transcriptional activators on the *TEF1+Z268* promoter and the *CYC1+Z268* promoter: cells in the exponential growth phase (**a**) and cells in the ethanol growth phase (**b**). The values were calculated from the data in Figure 1b. Mean values are shown (N = 3 independent biological replicates). Source data are provided as a Source Data file.

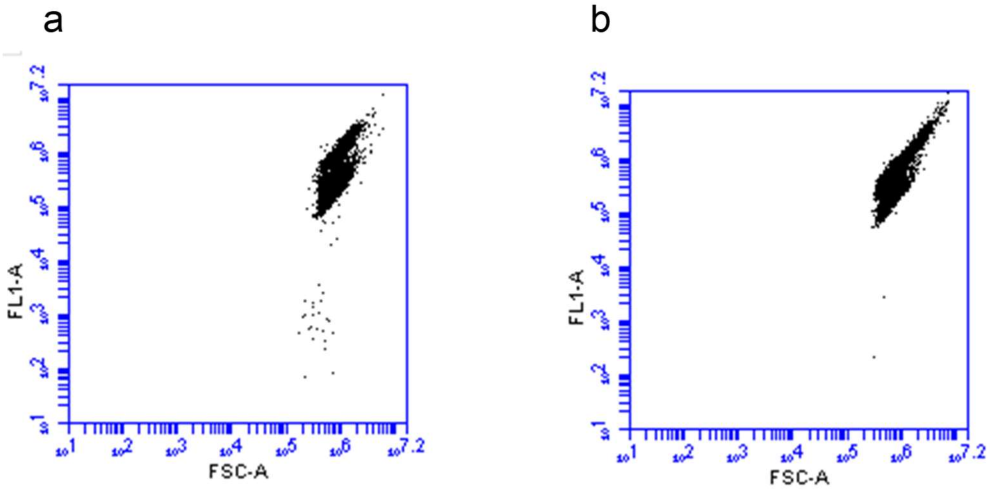

Supplementary Figure 2. Flow cytometry plots for strain G8H8M (**a**) expressing ZifPZ42-Med15 *trans*-activator and strain G8H8O (**b**) expressing ZifPZ43-Med15 *trans*-activator. The values represent the data from one of biological replicates.

Z268 elements: CTACCTGAATCTAAGATTGGGCCC
GCGTGGGCGaattggt**GCGTGGGCG**taagcgggggcgg**GCGTGGGCG**ta**GCGTGGGCG**
GCCGGCAATCCTCGAGCA

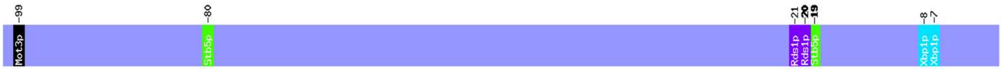

Mot3p	CAGGYA	-99	R
Skn7p	GGCYGGC	-19	F
Skn7p	GGCCGGC	-19	F
Skn7p	GGCCR	-19	F
Skn7p	GGCCR	-20	R
Stb5p	CGGNS	-20	F
Stb5p	CGGNS	-19	R
Stb5p	CGGNS	-80	R
Xbp1p	CTCGA	-8	F
Xbp1p	CTCGA	-7	R
Rds1p	BCGGCCG	-21	F
Rds1p	BCGGCCG	-20	R

PZ4 elements: CTACCTGAATCTAAGATTGGGCCCTA
GAGTGAGACGTTaattgg**tAGAGTGAGACGTT**taagcgggggcggC**TAGAGTGAGACGTT**gggct**taGAGT GAGACGTT**
GCCGGCAATCCTCGAGCA

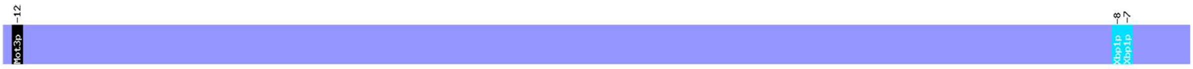

Mot3p	CAGGYA	-122	R
Xbp1p	CTCGA	-8	F
Xbp1p	CTCGA	-7	R

Supplementary Figure 3. Analysis of transcriptional factor (TF) binding sites in Z268/PZ4 element-containing sequences. TF binding sites were analysed through YEASTRACT web services (http://www.yeastract.com/formtfsbindingsites.php).

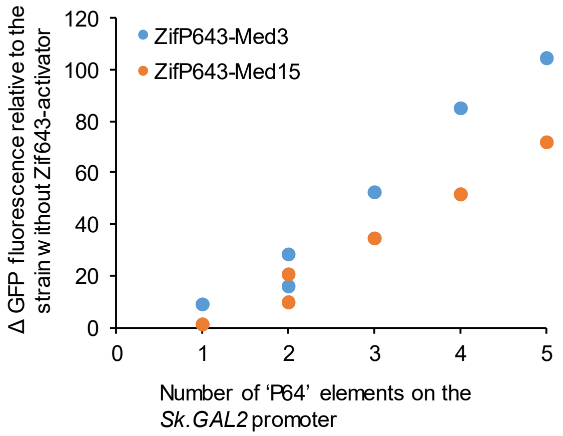

Supplementary Figure 4. The correlation of number of PZ4 elements in the *Sk.GAL2* promoter and the *trans*-activating effects by artificial *trans*-activators ZifPZ43-Med3 and ZifPZ43-Med15. The values were calculated from the data in Figure 2f. Mean values are shown (N = 3 independent biological replicates). Source data are provided as a Source Data file.

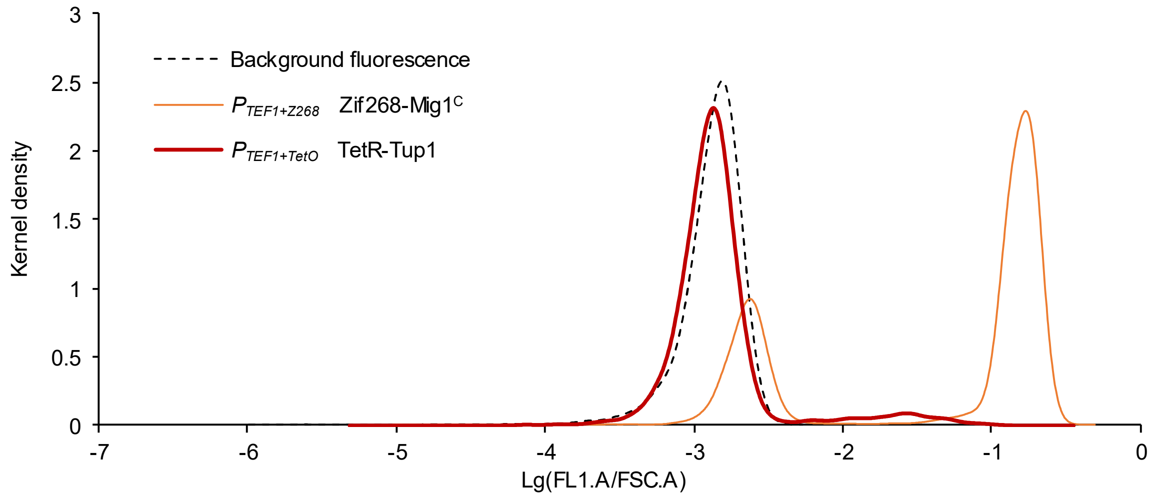

Supplementary Figure 5. Analysis of yEGFP fluorescence in strain G4D42J ($P_{TEF1+Z268}$ Zif268-Mig1^{C}: expressing yEGFP under the control of $P_{TEF1+Z268}$ and expressing Zif268-Mig1^{C} repressor) and strain G9E3S ($P_{TEF1+TetO}$ TetR-Tup1: expressing yEGFP under the control of $P_{TEF1+TetO}$ and expressing TetR-Tup1repressor). Strain GH4 [6] was used for background fluorescence analysis. Kernel density was calculated with the bandwidth of 0.05. The values represent the data from one of biological replicates. Source data are provided as a Source Data file.

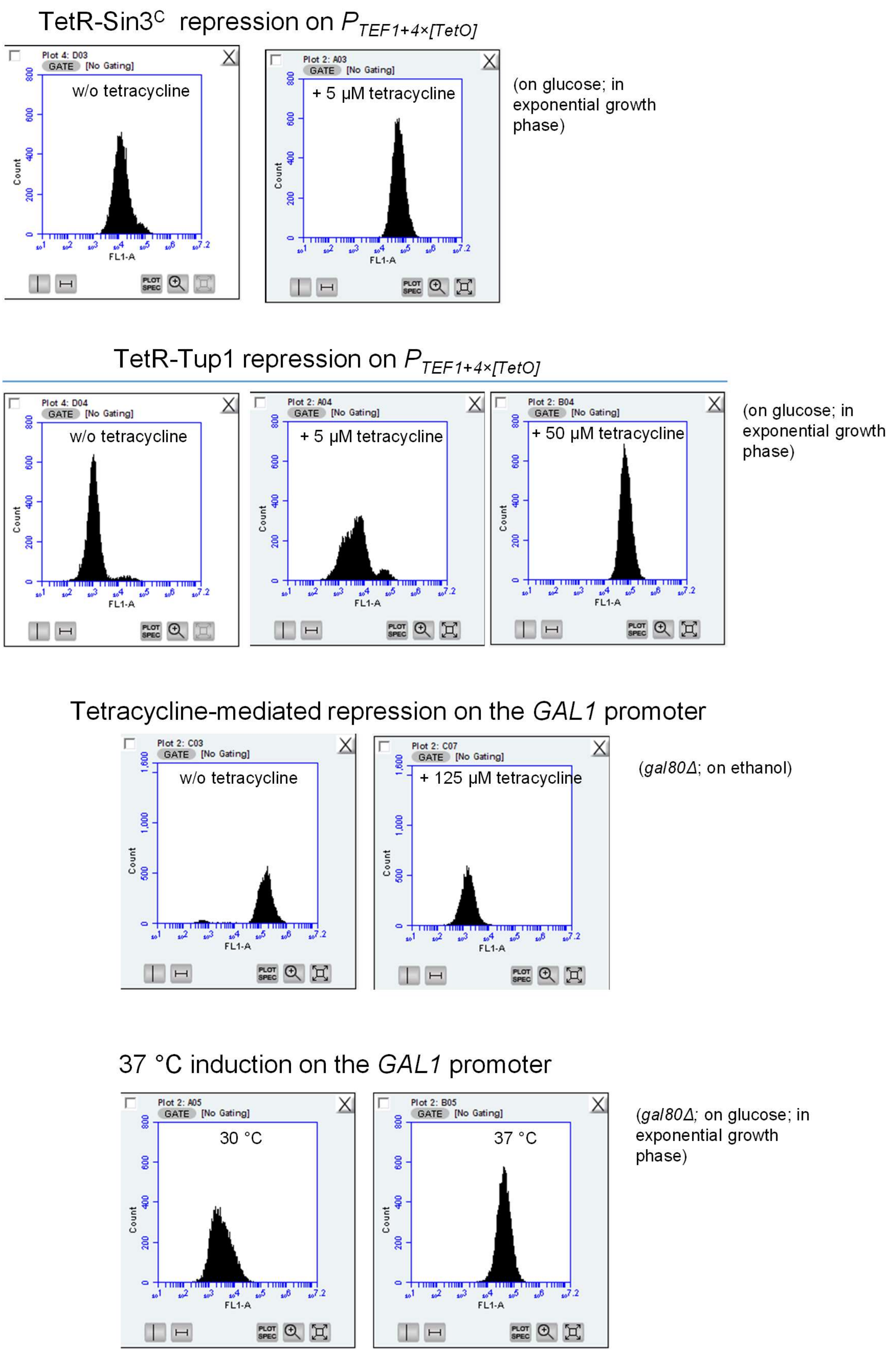

Supplementary Figure 6. Flow cytometry analysis of the population of yeast cells for tetracycline- and heat- responsive circuits. The profiles are screenshots from the analysis using BD Csampler software and represent the data from one of biological replicates.

Supplementary References

1. Lu, Z., Peng, B., Ebert, B.E., Dumsday, G. & Vickers, C.E. Auxin-mediated protein depletion for metabolic engineering in terpene-producing yeast. *Nature communications* **12**, 1051 (2021).
2. McIsaac, R.S., Gibney, P.A., Chandran, S.S., Benjamin, K.R. & Botstein, D. Synthetic biology tools for programming gene expression without nutritional perturbations in *Saccharomyces cerevisiae*. *Nucleic acids research* **42**, e48 (2014).
3. Sikorski, R.S. & Hieter, P. A System of Shuttle Vectors and Yeast Host Strains Designed for Efficient Manipulation of DNA in Saccharomyces-Cerevisiae. *Genetics* **122**, 19-27 (1989).
4. Güldener, U., Heck, S., Fielder, T., Beinhauer, J. & Hegemann, J.H. A new efficient gene disruption cassette for repeated use in budding yeast. *Nucleic acids research* **24**, 2519-2524 (1996).
5. Peng, B., Wood, R.J., Nielsen, L.K. & Vickers, C.E. An Expanded Heterologous GAL Promoter Collection for Diauxie-Inducible Expression in Saccharomyces cerevisiae. *ACS synthetic biology* (2018).
6. Peng, B., Williams, T., Henry, M., Nielsen, L. & Vickers, C. Controlling heterologous gene expression in yeast cell factories on different carbon substrates and across the diauxic shift: a comparison of yeast promoter activities. *Microbial cell factories* **14**, 91 (2015).
7. Gueldener, U., Heinisch, J., Koehler, G.J., Voss, D. & Hegemann, J.H. A second set of loxP marker cassettes for Cre-mediated multiple gene knockouts in budding yeast. *Nucleic acids research* **30**, e23 (2002).
8. Entian, K.D. & Kötter, P. in Yeast gene analysis. (eds. J.P.A. Brown & M.F. Tuite) (Academic Press, USA; 1998).
9. Peng, B., Plan, M.R., Carpenter, A., Nielsen, L.K. & Vickers, C.E. Coupling gene regulatory patterns to bioprocess conditions to optimize synthetic metabolic modules for improved sesquiterpene production in yeast. *Biotechnology for biofuels* **10**, 43 (2017).
10. Ellis, T., Wang, X. & Collins, J.J. Diversity-based, model-guided construction of synthetic gene networks with predicted functions. *Nature biotechnology* **27**, 465-471 (2009).
11. Aranda-Diaz, A., Mace, K., Zuleta, I., Harrigan, P. & El-Samad, H. Robust Synthetic Circuits for Two-Dimensional Control of Gene Expression in Yeast. *ACS synthetic biology* **6**, 545-554 (2017).
12. Camara, E., Lenitz, I. & Nygard, Y. A CRISPR activation and interference toolkit for industrial Saccharomyces cerevisiae strain KE6-12. *Scientific reports* **10**, 14605 (2020).
13. Stanton, B.C. et al. Genomic mining of prokaryotic repressors for orthogonal logic gates. *Nature chemical biology* **10**, 99-105 (2014).
14. Auslander, S. & Fussenegger, M. From gene switches to mammalian designer cells: present and future prospects. *Trends in biotechnology* **31**, 155-168 (2013).
15. Ikushima, S., Zhao, Y. & Boeke, J.D. Development of a Tightly Controlled Off Switch for Saccharomyces cerevisiae Regulated by Camphor, a Low-Cost Natural Product. *G3-Genes Genom Genet* **5**, 1983-1990 (2015).
16. Ikushima, S. & Boeke, J.D. New Orthogonal Transcriptional Switches Derived from Tet Repressor Homologues for Saccharomyces cerevisiae Regulated by 2,4-Diacetylphloroglucinol and Other Ligands. *ACS synthetic biology* **6**, 497-506 (2017).
17. Nakada, Y., Jiang, Y., Nishijyo, T., Itoh, Y. & Lu, C.-D. Molecular Characterization and Regulation of theaguBA Operon, Responsible for Agmatine Utilization inPseudomonas aeruginosa PAO1. *Journal of bacteriology* **183**, 6517-6524 (2001).
18. Sanchez, P., Alonso, A. & Martinez, J.L. Cloning and characterization of SmeT, a repressor of the Stenotrophomonas maltophilia multidrug efflux pump SmeDEF. *Antimicrobial agents and chemotherapy* **46**, 3386-3393 (2002).
19. Yoshida, K.-i. et al. Bacillus subtilis LmrA Is a Repressor of the lmrAB and yxaGH Operons: Identification of Its Binding Site and Functional Analysis of lmrB and yxaGH. *Journal of bacteriology* **186**, 5640-5648 (2004).
20. Mullick, A. et al. The cumate gene-switch: a system for regulated expression in mammalian cells. *BMC biotechnology* **6** (2006).

21. Abbas, A. et al. Characterization of Interactions between the Transcriptional Repressor PhlF and Its Binding Site at the phlA Promoter in Pseudomonas fluorescens F113. *Journal of bacteriology* **184**, 3008-3016 (2002).
22. Ikushima, S., Zhao, Y. & Boeke, J.D. Development of a Tightly Controlled Off Switch for Saccharomyces cerevisiae Regulated by Camphor, a Low-Cost Natural Product. *G3* **5**, 1983-1990 (2015).
23. Spiridonov, N.A. & Wilson, D.B. Characterization and cloning of celR, a transcriptional regulator of cellulase genes from Thermomonospora fusca. *The Journal of biological chemistry* **274**, 13127-13132 (1999).
24. Danot, O. & Raibaud, O. On the puzzling arrangement of the asymmetric MalT-binding sites in the MalT-dependent promoters. *Proceedings of the National Academy of Sciences of the United States of America* **90**, 10999-11003 (1993).
25. Wang, H. & Stillman, D.J. Transcriptional repression in Saccharomyces cerevisiae by a SIN3-LexA fusion protein. *Molecular and cellular biology* **13**, 1805-1814 (1993).
