# Supplementary material for: Engineering eukaryote-like regulatory circuits to expand artificial control mechanisms for metabolic engineering in Saccharomyces cerevisiae
Source: Commun Biol. 2022 Feb 16;5:135. doi: 10.1038/s42003-022-03070-z (PMC8850539; doi:10.1038/s42003-022-03070-z)
Supplement: Supplementary file 3 — Description of Additional Supplementary Files [file 42003_2022_3070_MOESM3_ESM.pdf]

Description of Additional Supplementary Files

File name: Supplementary Data 1
Description: Source data.
