# Supplementary material for: Engineering eukaryote-like regulatory circuits to expand artificial control mechanisms for metabolic engineering in Saccharomyces cerevisiae
Source: Commun Biol. 2022 Feb 16;5:135. doi: 10.1038/s42003-022-03070-z (PMC8850539; doi:10.1038/s42003-022-03070-z)
Supplement: Supplementary file 5 — Reporting Summary [file 42003_2022_3070_MOESM5_ESM.pdf]

nature research

Corresponding author(s): Bingyin Peng; Claudia Vickers

Last updated by author(s): Nov 3, 2021

Reporting Summary

Nature Research wishes to improve the reproducibility of the work that we publish. This form provides structure for consistency and transparency in reporting. For further information on Nature Research policies, see our Editorial Policies and the Editorial Policy Checklist.

Statistics

For all statistical analyses, confirm that the following items are present in the figure legend, table legend, main text, or Methods section.

n/a	Confirmed	
☐	☒	The exact sample size (*n*) for each experimental group/condition, given as a discrete number and unit of measurement
☐	☒	A statement on whether measurements were taken from distinct samples or whether the same sample was measured repeatedly
☐	☒	The statistical test(s) used AND whether they are one- or two-sided<br>*Only common tests should be described solely by name; describe more complex techniques in the Methods section.*
☒	☐	A description of all covariates tested
☒	☐	A description of any assumptions or corrections, such as tests of normality and adjustment for multiple comparisons
☐	☒	A full description of the statistical parameters including central tendency (e.g. means) or other basic estimates (e.g. regression coefficient) AND variation (e.g. standard deviation) or associated estimates of uncertainty (e.g. confidence intervals)
☐	☒	For null hypothesis testing, the test statistic (e.g. *F*, *t*, *r*) with confidence intervals, effect sizes, degrees of freedom and *P* value noted<br>*Give P values as exact values whenever suitable.*
☒	☐	For Bayesian analysis, information on the choice of priors and Markov chain Monte Carlo settings
☒	☐	For hierarchical and complex designs, identification of the appropriate level for tests and full reporting of outcomes
☒	☐	Estimates of effect sizes (e.g. Cohen's *d*, Pearson's *r*), indicating how they were calculated

Our web collection on statistics for biologists contains articles on many of the points above.

Software and code

Policy information about availability of computer code

Data collection	Data for HPLC was collected in Metabolomics Australia (Queensland node). Metabolomics Australia (Queensland node) used Agilent HPLC software, and Thermo Fisher Chromeleon Chromatography Data System softwares to collect data. We used a BD Csampler software (BD Accuri C6 software version 1.0.264.21) to collect Flow Cytometry data through BD Accuri C6 Flow Cytometry.
Data analysis	Metabolomics Australia (Queensland node) used an Agilent HPLC software to process HPLC data. We used Microsoft Office for data analysis.

For manuscripts utilizing custom algorithms or software that are central to the research but not yet described in published literature, software must be made available to editors and reviewers. We strongly encourage code deposition in a community repository (e.g. GitHub). See the Nature Research guidelines for submitting code & software for further information.

Data

Policy information about availability of data

All manuscripts must include a data availability statement. This statement should provide the following information, where applicable:

- Accession codes, unique identifiers, or web links for publicly available datasets
- A list of figures that have associated raw data
- A description of any restrictions on data availability

Source data are provided with this paper.

Field-specific reporting

Please select the one below that is the best fit for your research. If you are not sure, read the appropriate sections before making your selection.

☒ Life sciences ☐ Behavioural & social sciences ☐ Ecological, evolutionary & environmental sciences

For a reference copy of the document with all sections, see nature.com/documents/nr-reporting-summary-flat.pdf

Life sciences study design

All studies must disclose on these points even when the disclosure is negative.

Sample size	Sample size was not calculated through a statistical method. Sample sizes were chosen on an empirical or pragmatical base in the field of metabolic engineering and synthetic biology in yeast. The sample size for construction-in-processing strains is one, when we hypothesize that the spontaneous genetic mutation is insignificant and we can confirm the genotype. The samples size for final characterization strains are at least two.
Data exclusions	No data were excluded.
Replication	Characterization of final yeast strains were performed with biological replicates: two-to-four biological replicates were performed in Figure 1, Figure 2, Figure 3, Figure 4, Figure 5, Supplementary Figure 1, and Supplementary Figure 4. In Supplementary Figure 2, supplementary Figure 5 and Supplementary Figure 6, the data from one of biological replicates were presented. We did not execute the replication experiment for characterization of construction-in-processing strains and strain construction procedures.
Randomization	The biological replicates were chosen randomly.
Blinding	Not applicable, due to authors collaterally designed, performed experiments, and collected data in the current study.

Reporting for specific materials, systems and methods

We require information from authors about some types of materials, experimental systems and methods used in many studies. Here, indicate whether each material, system or method listed is relevant to your study. If you are not sure if a list item applies to your research, read the appropriate section before selecting a response.

Materials & experimental systems

n/a	Involved in the study	
☒	☐	Antibodies
☒	☐	Eukaryotic cell lines
☒	☐	Palaeontology and archaeology
☒	☐	Animals and other organisms
☒	☐	Human research participants
☒	☐	Clinical data
☒	☐	Dual use research of concern

Methods

n/a	Involved in the study	
☒	☐	ChIP-seq
☐	☒	Flow cytometry
☒	☐	MRI-based neuroimaging

Flow Cytometry

Plots

Confirm that:

☒ The axis labels state the marker and fluorochrome used (e.g. CD4-FITC).

☒ The axis scales are clearly visible. Include numbers along axes only for bottom left plot of group (a 'group' is an analysis of identical markers).

☒ All plots are contour plots with outliers or pseudocolor plots.

☒ A numerical value for number of cells or percentage (with statistics) is provided.

Methodology

Sample preparation	Samples for fluorescence analysis in Yeast cells were directly used for flow cytometry analysis. Samples for analyzing Y-FAST fluorescence in yeast cells were used for flow cytometry analysis after chromophore was added. The details have been described in Methods.

Instrument	Accuri C6 plus
Software	Accuri C6 plus sampler
Cell population abundance	10,000 events are analysed for each data point in this study.
Gating strategy	FSC thresholding was used to exclude small debris particles during data collection. Other gating strategy was not used. All collected events were included in the analysis.

☐ Tick this box to confirm that a figure exemplifying the gating strategy is provided in the Supplementary Information.
